# Rice F-bZIP transcription factors regulate the zinc deficiency response

**DOI:** 10.1093/jxb/eraa115

**Published:** 2020-03-05

**Authors:** Grmay H Lilay, Pedro Humberto Castro, Joana G Guedes, Diego M Almeida, Ana Campilho, Herlander Azevedo, Mark G M Aarts, Nelson J M Saibo, Ana G L Assunção

**Affiliations:** 1 Department of Plant and Environmental Sciences, Copenhagen Plant Science Centre, University of Copenhagen, Frederiksberg-C, Denmark; 2 CIBIO-InBIO, Research Centre in Biodiversity and Genetic Resources, Campus Agrário de Vairão, University of Porto, Vairão, Portugal; 3 Genomics of Plant Stress Laboratory, Instituto de Tecnologia Química e Biológica António Xavier, New University of Lisbon, Oeiras, Portugal; 4 Department of Biology, Faculty of Sciences, University of Porto, Porto, Portugal; 5 Laboratory of Genetics, Wageningen University& Research, Wageningen, The Netherlands; 6 Biology Center of the Czech Academy of Sciences, Czech Republic

**Keywords:** Biofortification, F-bZIP, monocots, phylogenetic analysis, rice (*Oryza sativa*), zinc deficiency, ZIP transporters

## Abstract

The F-bZIP transcription factors bZIP19 and bZIP23 are the central regulators of the zinc deficiency response in Arabidopsis, and phylogenetic analysis of F-bZIP homologs across land plants indicates that the regulatory mechanism of the zinc deficiency response may be conserved. Here, we identified the rice F-bZIP homologs and investigated their function. *OsbZIP48* and *OsbZIP50*, but not *OsbZIP49*, complement the zinc deficiency-hypersensitive Arabidopsis *bzip19bzip23* double mutant. Ectopic expression of *OsbZIP50* in Arabidopsis significantly increases plant zinc accumulation under control zinc supply, suggesting an altered Zn sensing in OsbZIP50. In addition, we performed a phylogenetic analysis of F-bZIP homologs from representative monocot species that supports the branching of plant F-bZIPs into Group 1 and Group 2. Our results suggest that regulation of the zinc deficiency response in rice is conserved, with OsbZIP48 being a functional homolog of AtbZIP19 and AtbZIP23. A better understanding of the mechanisms behind the Zn deficiency response in rice and other important crops will contribute to develop plant-based strategies to address the problems of Zn deficiency in soils, crops, and cereal-based human diets.

## Teaser

Zinc deficiency in soils and crops is a global problem. We combine functional and phylogenetic analyses to unravel the role of rice F-bZIP homologs in the zinc deficiency response.

## Introduction

Zinc (Zn) is an essential micronutrient for all organisms because of its catalytic and structural roles in many proteins, with Zn-binding proteins estimated at ~10% of eukaryote proteomes (Andreini *et al.*, 2006). Zn-deficient soils are widespread globally, particularly in large parts of Africa and Asia, affecting yield and nutritional quality of crops (Alloway, 2008). Rice (*Oryza sativa* L.) is one of the most important food crops worldwide, feeding nearly half of the world’s population, especially in Asia, as the staple food in many areas, but it does not provide enough essential mineral nutrients to match human requirements (Welch and Graham, 2004; Nakandalage *et al.*, 2016). The risk of Zn deficiency is estimated to affect about one-third of the world’s human population, and it can lead to different degrees of growth retardation, immune dysfunction, and cognitive impairment (Prasad, 2009; Wessells and Brown, 2012). Improving Zn use efficiency and Zn accumulation in the edible parts of crops (Zn biofortification), in combination with agronomic strategies, constitute plant-based solutions to tackle these global problems (Cakmak, 2002; White and Broadley, 2009).

A better understanding of the molecular mechanisms of plant response to Zn deficiency can contribute to improve traits in crops such as Zn use efficiency, Zn accumulation, and adaptation to Zn-deficient soils. In the model plant *Arabidopsis thaliana* (Arabidopsis), the bZIP19 and bZIP23 transcription factors are the central regulators of the Zn deficiency response (Assunção *et al.*, 2010). They are members of the F group of Arabidopsis basic-leucine zipper proteins (F-bZIPs), characterized by the presence of a region, at the N-terminus, rich in cysteine and histidine residues (Cys/His-rich motif). The third F-bZIP member is bZIP24, involved in regulation of salt stress tolerance (Jakoby *et al.*, 2002; Yang *et al.*, 2009). The *bzip19bzip23* double mutant (*bzip19/23*) is hypersensitive to Zn deficiency, but has no visible phenotype under Zn sufficiency. The bZIP19 and bZIP23 transcription factors are localized to the nucleus and bind to a 10 bp *Zinc Deficiency Response Element* (*ZDRE*; RTGTCGACAY) that is present in the promoter region of their target genes (Assunção *et al.*, 2010). Under Zn deficiency, bZIP19 and bZIP23, which are partially redundant, activate transcription of a small set of Zn homeostasis genes, involved in Zn transport and distribution. These include genes encoding ZIP (Zinc-regulated/Iron-regulated Protein) family Zn transporters involved in cellular Zn uptake, and genes encoding NAS (nicotianamine synthase) enzymes that produce the Zn chelator nicotianamine (NA) involved in Zn distribution (Guerinot, 2000; Assunção *et al.*, 2010; Clemens *et al.*, 2013; Inaba *et al.*, 2015).

A phylogenetic analysis of F-bZIP homologs across land plants suggested conservation of the Zn deficiency response regulatory mechanism. It also showed that predicted orthologs of the AtbZIP19 and AtbZIP23 target *ZIP* genes, from representative land plant species, also contain *ZDRE* motifs in their promoters (Castro *et al.*, 2017). This is supported by functional analysis of F-bZIP members from wheat (*Triticum aestivum*) and barley (*Hordeum vulgare*) (Evens *et al.*, 2017; Nazri *et al.*, 2017). In barley, seven F-bZIP homologs were identified, of which HvbZIP56 and HvbZIP62 complemented the Arabidopsis *bzip19/23* mutant Zn deficiency phenotype. The complemented line with HvbZIP56 was also shown to rescue AtbZIP19/23 *ZIP* target gene regulation, and subcellular localization analysis of this protein indicated nuclear and cytosolic expression (Nazri *et al.*, 2017). In wheat, 21 F-bZIP homologs, corresponding to seven groups with homeologs from A, B, and D genomes were identified, of which TabZIPF1-7DL and TabZIPF4-7AL rescue the *bzip19/23* Zn deficiency phenotype. The TabZIPF1-7DL protein was also shown to bind to the *ZDRE* motif (Evens *et al.*, 2017). In addition, ZDRE motifs were identified in the promoters of barley and wheat *ZIP* genes, overall supporting the conservation of the function of F-bZIPs in the Zn deficiency response regulatory network (Castro *et al.*, 2017; Evens *et al.*, 2017; Nazri *et al.*, 2017). Also, in *Brachypodium distachyon*, the F-bZIP BdbZIP10 is suggested to be involved in regulation of Zn homeostasis (Martin *et al.*, 2018). Considering the global importance of rice as a staple food crop, especially in areas with prevalence of Zn-deficient human diets, we performed the functional characterization of the rice F-bZIP homologs to uncover their role in the Zn deficiency response. We also performed a detailed phylogenetic analysis with F-bZIP homologs retrieved from representative monocot species. A better understanding of the mechanisms behind the Zn deficiency response in rice and in other important crops will contribute to effectively address Zn use efficiency and Zn biofortification in crops.

## Materials and methods

### Phylogenetic and synteny analysis

Phylogeny of monocot *F-bZIP* genes was determined as previously reported (Castro *et al.*, 2017). Amino acid sequences of monocot F-bZIPs were obtained from the comparative database Plaza Monocots 4.5 (Van Bel *et al.*, 2018) for the following species: *Ananas comosus*, *Brachypodium distachyon*, *Elaeis guineensis*, *Hordeum vulgare*, *Musa acuminata*, *Oropetium thomaeum*, *Oryza brachyantha*, *Oryza sativa* ssp. *indica*, *Oryza sativa* ssp. *japonica*, *Phalaenopsis equestris*, *Phyllostachys edulis*, *Setaria italica*, *Sorghum bicolor*, *Spirodela polyrhiza*, *Triticum aestivum*, *Zea mays*, *Zostera marina*, and *Zoysia japonica* ssp. *nagirizaki*. For the monocot sequences we opted to use the most recent nomenclature available in Plaza Monocots 4.5. A highly incomplete sequence, and indicated as an outlier by the database, was found for *O. thomaeum* (Oropetium_20150105_23063) and therefore it was excluded from the analysis. To outgroup and have a better evolutionary perspective of the F-bZIPs across land plants, other species were selected as being representative of major plant taxa, including a bryophyte (*Physcomitrella pattens*), a pteridophyte (*Selaginella moellendorffii*), a gymnosperm (*Picea glauca*), the basal angiosperm (*Amborella trichopoda*), the model angiosperm eudicot (*Arabidopsis thaliana*), and another three dicot species containing one Group 1 and one Group 2 F-bZIP (*Prunus persica*, *Theobroma cacao*, and *Vitis vinifera*) (Castro *et al.*, 2017). Phylogenetic analysis was performed using CIPRES Science Gateway V3.3 (http://www.phylo.org) (Miller *et al.*, 2011) and computed inputting 1000 bootstrap iterations, as previously described (Castro *et al.*, 2017). The output tree was visualized in the SeaView Version 4 software (Gouy *et al.*, 2010). To resolve syntenic relationships, we used the colinear gene pairs option at Plaza Monocots 4.5 (Van Bel *et al.*, 2018) to identify colinear segments (multiplicons) associated with each F-bZIP gene present in a selected set of species (the above-mentioned monocots, and dicots *A. thaliana* and *A. trichopoda*). Information on multiplicon properties (#Archorpoints and Profile length) was retrieved for each pairwise gene comparison, and the ratio of #Archorpoints per Profile length was used as a measure of collinearity strength. The ratio was applied as the edge score for the construction of a network on Cytoscape (Shannon, 2003), using the yFiles_Organic layout and removing genes with no observed collinearity score. Edge thickness was set between the minimum (0.06) and maximum (0.75) observed scores.

### Plasmid construction and plant transformation

The *pCaMV35S::*bZIP-CFP-HA constructs for stable transformation of the Arabidopsis *bzip19/23* double mutant were generated as follows: the full-length coding sequences (CDSs) of *OsbZIP48* (LOC_Os06g50310), *OsbZIP49* (LOC_Os01g58760), and *OsbZIP50* (LOC_Os05g41540) were amplified from a rice (cv. Nipponbare) cDNA library using forward and reverse primers containing *Not*I and *Asc*I restriction sites, respectively (Supplementary Table S1 at *JXB* online). The cloning into pEarleyGate-102 Gateway vector (Earley *et al.*, 2006), carrying a *Cauliflower mosaic virus* (CaMV) *35S* promoter, a C-terminal cyan fluorescent protein (CFP), and a HA-tag, the transformation into *Agrobacterium tumefaciens*, and production of the Arabidopsis *bzip19/23* mutant were performed as described by Lilay *et al*. (2019). Transgenic plants were selected for Basta (phosphinothricin) resistance, and homozygous transgenic seeds (T_3_ generation) of three independent lines per construct were selected. The expression of each *F-bZIP* gene was confirmed in the respective lines by real-time quantitative reverse transcription–PCR (RT–qPCR). The lines were referred to as *bzip19/23*-OE*Os*48, *bzip19/23*-OE*Os*49, and *bzip19/23*-OE*Os*50.

### Plant material and growth conditions

For hydroponic growth, rice seeds (cv. Nipponbare) were heat treated in paper bags for 5 d at 50 °C and then germinated on vermiculite with distilled water, at 28 °C, for 48 h in the dark, followed by 5 d with a 12 h light/dark cycle and 70% relative humidity. One-week-old seedlings were transferred to 5 liter pots with a lid containing four holes, one for every seedling held supported by rock-wool. A Yoshida nutrient solution (Almeida *et al.*, 2016) buffered with 0.5 mM MES at pH 5.1 was used, with 0.15 µM ZnSO_4_ (control) or with no ZnSO_4_ added (–Zn). The plastic pots, for the control and –Zn treatments, were rinsed with 0.1 N HCl followed by five rinses with ultrapure water prior to use. The nutrient solutions were prepared with ultrapure water. Rice plants were grown for 12 weeks in either control or –Zn nutrient solution, which was aerated throughout the experiment, with four plants per pot and five pots per Zn treatment. The nutrient solutions were replaced once a week during the first 4 weeks, and twice in the weeks thereafter, with daily control and adjustment of pH. For the complementation analysis in Arabidopsis, the wild-type (accession Columbia, Col-0) and the *bzip19 bzip23* double mutant (*bzip19/*23; in the Col-0 background, described by Assunção *et al.*, 2010) were used in all experiments. The complementation analysis was part of a larger experimental set-up that simultaneously addressed complementation of *bzip19/23* with Arabidopsis F-bZIPs (Lilay *et al*., 2018) and rice F-bZIPs (this study), thus data for the wild-type and *bzip19/23*, positive and negative control genotypes, respectively, are common in both studies. For agar-grown seedlings, sterilized seeds of the wild type, *bzip19/23* mutant, and *bzip19/23*-OE*Os*48, *bzip19/23*-OE*Os*49, and *bzip19/23*-OE*Os*50 lines were sown on half-strength Murashige and Skoog (MS) medium containing 15 µM ZnSO_4_ (control) or no added Zn (–Zn) as described by Lilay *et al*. (2018). The lines were grown together in plates (~5 seedlings per genotype) with control or –Zn MS medium for 14 d. For each complementation line, three independently transformed transgenic lines were tested with at least four plates (replicates) per line and per Zn condition. For the analysis with hydroponically grown plants, sterilized seeds of the above-mentioned lines were germinated and grown for 8 weeks with a modified half-strength Hoagland nutrient solution with either 2 µM ZnSO_4_ (control) or 0.002 µM ZnSO_4_ (–Zn), with six plants per genotype, as described by Lilay *et al*. (2018). For the analysis with the soil-grown plants, 2-week-old seedlings of the above-mentioned lines, grown on control MS, were transplanted to a mixture of 80% peat–20% vermiculite, one seedling per 0.4 liter pot with six pots per line, and were grown for an additional 4 weeks. The hydroponics set-up and the MS plates with a 8/16 h light/dark cycle, and the pots with a 8/16 h and 16/8 h light/dark cycle were placed in a growth chamber, with 125 µmol m^−2^ s^−1^ white light, 22/20 °C light/dark temperature, and 70% relative humidity.

### Yeast one-hybrid (Y1H)assay

Total RNA was isolated from rice seedlings, as described by Almeida *et al.* (2017). The cDNA expression library was synthesized according to HybriZAP-2.1 XR cDNA Synthesis and Library Construction Kits (Stratagene), as described by the manufacturer. The screening was performed according to the Matchmaker, One-Hybrid System (Clontech). The reporter vectors containing bait F and bait G were introduced into yeast strain PJ69-4A, as previously reported (Assunção *et al.*, 2010). Yeast bait strains were transformed with 1 µg of the cDNA expression library. For each reporter vector, >1 million yeast colonies were screened in CM-His supplemented with 10–40 mM 3-aminotriazole (3-AT), as previously described (Ouwerkerk and Meijer, 2001). The identified clones were re-streaked on selective medium to confirm growth. Direct PCR on the yeast colonies was performed to amplify the cDNA insert, using specific primers for the library plasmid. The sequences were used to search for homology in the rice genome.

### EMSA

The full-length CDSs of *OsbZIP48* (LOC_Os06g50310) and *OsbZIP49* (LOC_Os01g58760) were amplified from a rice (cv. Nipponbare) cDNA library using forward and reverse primers containing *Not*I and *Asc*I restriction sites, respectively (Supplementary Table S1). The PCR products were purified with PureLink (Invitrogen) and cloned into the pENTR™/D-TOPO vector (Invitrogen) followed by *in vitro* site-directed recombination into the pSPUTK *in vitro* translation vector (Stratagene) using LR Clonase™ II Enzyme Mix (Invitrogen). All constructs were verified by restriction enzyme digestion analysis and DNA sequencing. The *in vitro* translation of *OsbZIP48* and *OsbZIP49*, the oligonucleotide labeling, and the EMSA were performed as described by Assunção *et al.* (2010).

### Real-time quantitative RT–PCR analysis

Roots and shoots from 4-week-old rice plants grown in control or –Zn hydroponic solution were separated and immediately frozen in liquid nitrogen in pools of two plants per pot and five pots per treatment. Roots and shoots were ground with liquid nitrogen using a mortar and pestle, and total RNA was extracted using the Direct-zol RNA Kit (Zymo Research). Fourteen-day-old Arabidopsis seedlings of the wild type, *bzip19/23* mutant, and *bzip19/23*-OE*Os*48, *bzip19/23*-OE*Os*49, and *bzip19/23*-OE*Os*50 lines, grown in control or –Zn MS medium, were harvested and immediately frozen in liquid nitrogen in pools of five seedlings per line and per Zn treatment×3 different plates grown simultaneously and considered as biological replicates. This experiment was performed twice, with two independently transformed T_3_ homozygous lines per genotype. Seedlings were ground with liquid nitrogen in a microtube, with the help of polypropylene pestles, and total RNA was extracted using the RNeasy Plant Mini Kit (Qiagen). RNA quantity and integrity, and cDNA synthesis were assessed and performed as described by Lilay *et al*. (2018). Primers for RT–qPCR (Supplementary Table S2) were designed using NCBI Primer-BLAST (www.ncbi.nlm.nih.gov/tools/primer-blast), and the primer amplification efficiency for each primer pair was between 1.9 and 2.1. RT–qPCR was performed with a LightCycler 96 Real-Time PCR System (Roche Diagnostics), using HOT FIREPol EvaGreen qPCR Mix (Solis BioDyne) in a 20 µl PCR mixture, as described by Lilay *et al*. (2018). Rice *Eukaryotic elongation factor1-alpha* (*OseEF-1α*, LOC_Os03g08020) and Arabidopsis *Actin-2* (*ACT2*, At3g18780) were used as reference genes in rice and Arabidopsis gene expression analysis, respectively. Reactions were performed in 2–3 technical replicas per biological replicate and in three biological replicates per line and Zn treatment. The calculated cycle threshold (Ct) value for each gene was normalized to the reference gene calculated Ct value (*OseEF-1α* for rice and *ACT2* for Arabidopsis). The relative transcript levels were expressed against the wild-type grown under control conditions, and calculated according to the 2^−ΔΔCT^ method (Livak and Schmittgen, 2001).

### Subcellular localization analysis

Ten-day-old seedlings of the wild type, *bzip19/23* mutant, and *bzip19/23*-OE*Os*48, *bzip19/23*-OE*Os*49, and *bzip19/23*-OE*Os*50 lines were grown in control or –Zn MS medium. For laser scanning confocal microscopy (LSCM) analysis, roots were transferred to microscope slides containing propidium iodide (PI) to stain root cell walls, and were visualized using a Leica TCS SP5 II laser scanning confocal inverted microscope (Leica Microsystems) with a HC PL APO CS ×63/1.30 Glycerine objective. Argon 458 nm and 514 nm laser lines were used for CFP and PI excitation, respectively. The emission settings were between 470 nm and 500 nm for CFP and 590 nm and 630 nm for PI. For each complementation line, three independently transformed T_3_ homozygous lines were tested, with observations of 2–3 seedlings per line and Zn condition.

### Tissue element analysis

For rice plants, shoots and roots from 4-week-old hydroponically grown plants (five plants per Zn treatment), grown in control or –Zn nutrient solution, were harvested. For Arabidopsis plants, shoots and roots from 8-week-old hydroponically grown plants from the wild type, *bzip19/23* mutant, and *bzip19/23*-OE*Os*48, *bzip19/23*-OE*Os*49, and *bzip19/23*-OE*Os*50 lines (four plants per line per Zn treatment), grown in control or –Zn nutrient solution, were harvested. For the *bzip19/23* mutant and *bzip19/23*-OE*Os*49 lines grown at –Zn, six plants were harvested, following the same procedure, and were analyzed in pools of two. This experiment was performed twice, with two or three independently transformed T_3_ homozygous lines per genotype. Arabidopsis plants from *bzip19/23*-OE*Os*48, *bzip19/23*-OE*Os*49, and *bzip19/23*-OE*Os*50 were also grown on soil, and the shoots from four plants per line were harvested. Shoot and root harvest, tissue digestion, and multielemental analysis, using inductively-coupled plasma mass spectrometry (ICP-MS) (7900 ICP-MS, Agilent Technologies), were performed as described by Lilay *et al*. (2018).

### Statistical analysis

To compare lines or treatments, we used one-way ANOVA followed by Tukey’s post-hoc test, or Student’s *t*-test, as appropriate, calculated with IBM SPSS Statistics V22.0 software.

## Results

### Yeast-one-hybrid screening identifies rice F-bZIPs

**Fig. 1.**
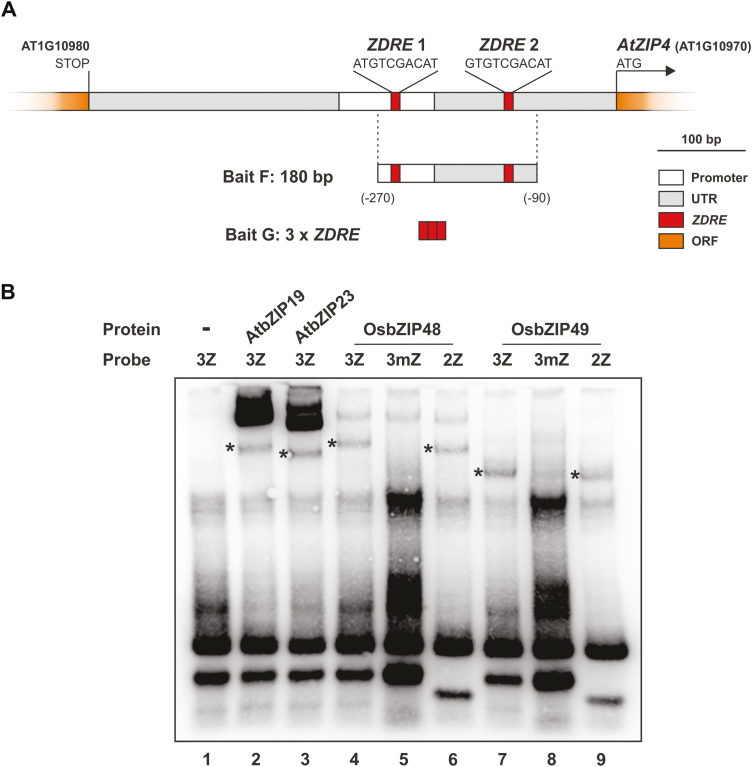
Yeast-one-hybrid (Y1H) screening and EMSA analysis. (A) Schematic diagram of the bait fragments (F and G) used to construct the reporter vectors in the Y1H assay. (B) EMSA analysis with *in vitro* translated OsbZIP48, OsbZIP49, and, as reference, AtbZIP19 and AtbZIP23. Probe acronyms 2Z and 3Z correspond to two or three *ZDRE* (ATGTCGACAY) tandem repeats, respectively, and 3mZ corresponds to three tandem repeats of a *ZDRE* mutated version (ATGT**A**GACAY).

To identify rice transcription factors that bind to the *ZDRE* promoter element, we performed a Y1H screening. As bait we employed either a three tandem repeat of the *ZDRE* motif (RTGTCGACAY) (Fig. 1A, Bait F), or a 180 bp promoter fragment of the *AtZIP4* Zn transporter gene (fragment G) that contains two *ZDRE* copies (Assunção *et al.*, 2010) (Fig. 1A, Bait G). The corresponding yeast bait strains were screened with a rice cDNA expression library, resulting in the identification of LOC_Os06g50310 (one clone) and LOC_Os01g58760 (two clones) as binding to bait F, and LOC_Os06g50310 (two clones) as binding to bait G (Supplementary Table S3). These loci correspond to the rice F-bZIP members, OsbZIP48 and OsbZIP49, respectively.

To validate the binding ability of OsbZIP48 and OsbZIP49 to the *ZDRE* motif, we carried out an *in vitro* binding assay using EMSA. Two and three tandem copies of *ZDRE* were used, and also three tandem copies of a modified *ZDRE* motif in which the TCGA core has been mutated to TAGA (RTGT**A**GACAY). The EMSA showed that both OsbZIP48 and OsbZIP49 can bind *in vitro* to three and two tandem copies of the *ZDRE* motif, but not to three tandem copies of the modified *ZDRE* version (Fig. 1B).

### Annotated F-bZIP homologs in rice and phyl.ogenetic analysis of monocot F-bZIPs

Previously, we carried out a thorough phylogenetic characterization of F-bZIP homologs across land plants that identified the presence of three F-bZIP members in rice, namely LOC_Os06g50310, LOC_Os01g58760, and LOC_Os05g41540, which correspond to OsbZIP48, OsbZIP49, and OsbZIP50, respectively. OsbZIP48 was mapped to F-bZIP Group 1, together with Arabidopsis bZIP19 and bZIP23, whereas OsbZIP49 and OsbZIP50 were included in Group 2, together with Arabidopsis bZIP24 (Castro *et al.*, 2017). The protein sequence alignments show a high amino acid sequence identity between the rice and Arabidopsis F-bZIP homologs (Supplementary Fig. S1A, B). Notably, the OsbZIP49 protein corresponds to a form truncated at the N-terminal end (Supplementary Fig. S1B).

**Fig. 2.**
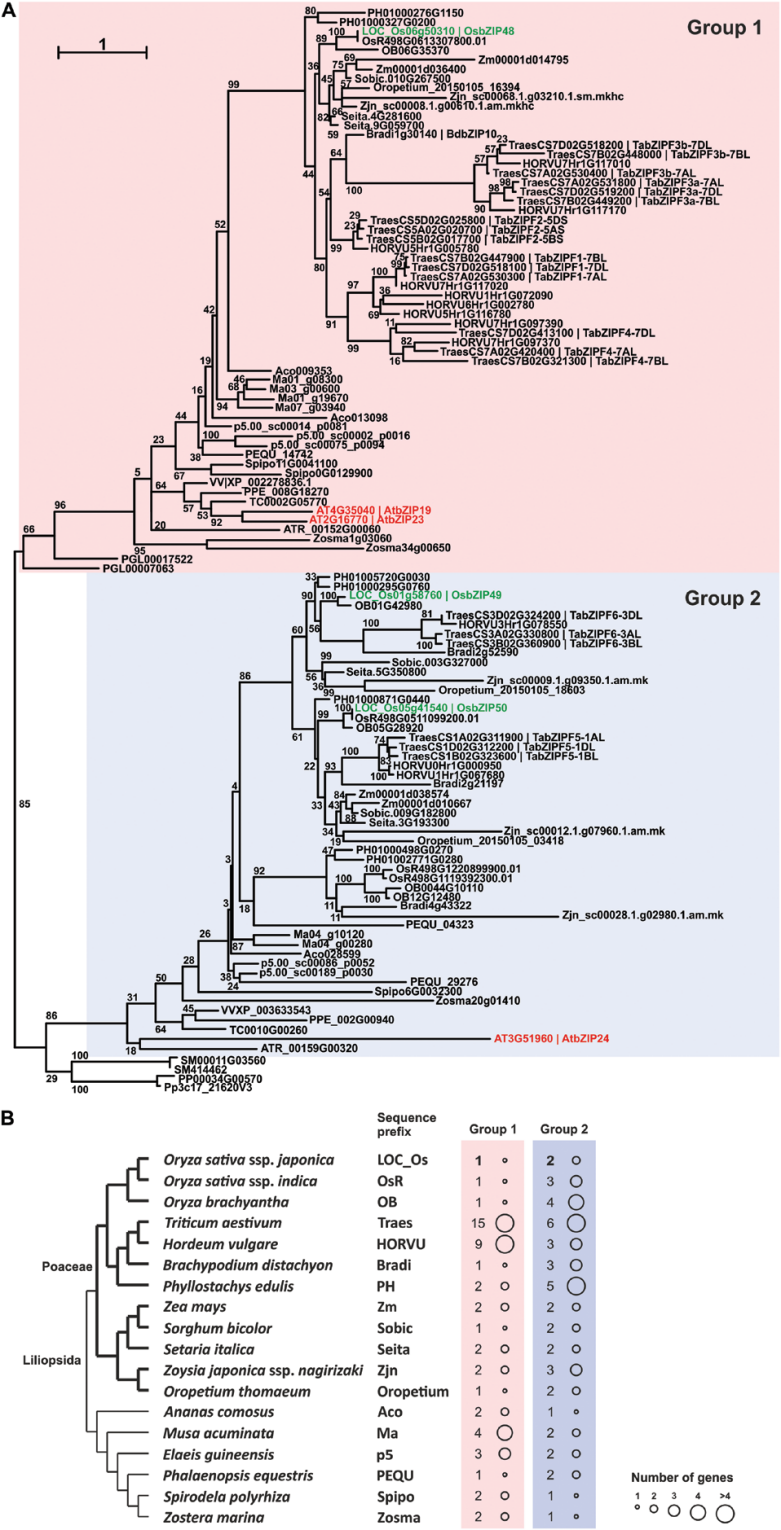
Phylogenetic analysis of the F-bZIP proteins in monocot plant species. (A) Phylogenetic tree representing 17 monocot species, the major taxa within monocots, plus representative species from other major plant taxa. The tree was constructed using maximum likehood and bootstrap values from 1000 replicates. Numbers on each branch represent the bootstrap percentages. (B) F-bZIP gene enrichment for each monocot plant species. Circle size represents the absolute number of expected genes present in the genome of each species.

In our previous phylogenetic analysis (Castro *et al.*, 2017), a monocot enrichment for Group 2 genes was suggested. Here, in order to obtain a more detailed evolutionary perspective of the monocot F-bZIP homologs, we increased resolution with the incorporation of additional genomes. Towards this end, a set of monocot species representative of the taxon’s phylogeny were selected. In total, 98 monocot F-bZIP sequences were identified (Supplementary Table S4), characterized by the presence of the bZIP domain and the F-bZIP characteristic Cys/His-rich motif, though truncated versions were observed. The full protein sequences were subsequently used for phylogenetic inference (Fig. 2A). Previously, we demonstrated that F-bZIP Group 1 and 2 emergence was associated with seed plant differentiation (Castro *et al.*, 2017). Here, we once again observed that bryophyte and pteridophyte sequence positioning supports a single monophyletic origin for F-bZIP transcription factors, with differentiated branches forming Group 1 and Group 2 F-bZIPs. In this report, we introduced a collinearity analysis across F-bZIP members to further establish the robustness of Group 1 and Group 2 differentiation (Supplementary Fig. S2). This analysis serves as a survey to infer the evolution of gene family members, complementing phylogeny by looking at paralogs within their genomic context. Results showed no collinearity between Group 1 and 2 genes, but strong collinearity within group members, which supports the phylogenetic analysis in Fig. 2A.

In addition to the rice subspecies *japonica* F-bZIP members, we identified the F-bZIPs from subspecies *indica*, with OsR498G0613307800.01 mapped to Group 1 and OsR498G0511099200.01, OsR498G1119392300.01, and OsR498G1220899900.01 mapped to Group 2. Given the diversity of species employed in this phylogenetic analysis, it supports the previously observed enrichment of Group 2 F-bZIP monocots (Castro *et al.*, 2017), in relation to Group 1, with the exception of wheat and barley (Fig. 2A), and indicates that the enrichment was confined to the Poaceae family, and was absent from the pineapple genome at the basis of the Poales order (Fig. 2A, B). In addition, Fig. 2B shows that all analyzed monocot species have at least one Group 1 F-bZIP member, in support of previous findings across land plant species (Castro *et al.*, 2017).

### Expression of rice *F-bZIP* and *ZIP* genes in response to Zn supply

**Fig. 3.**
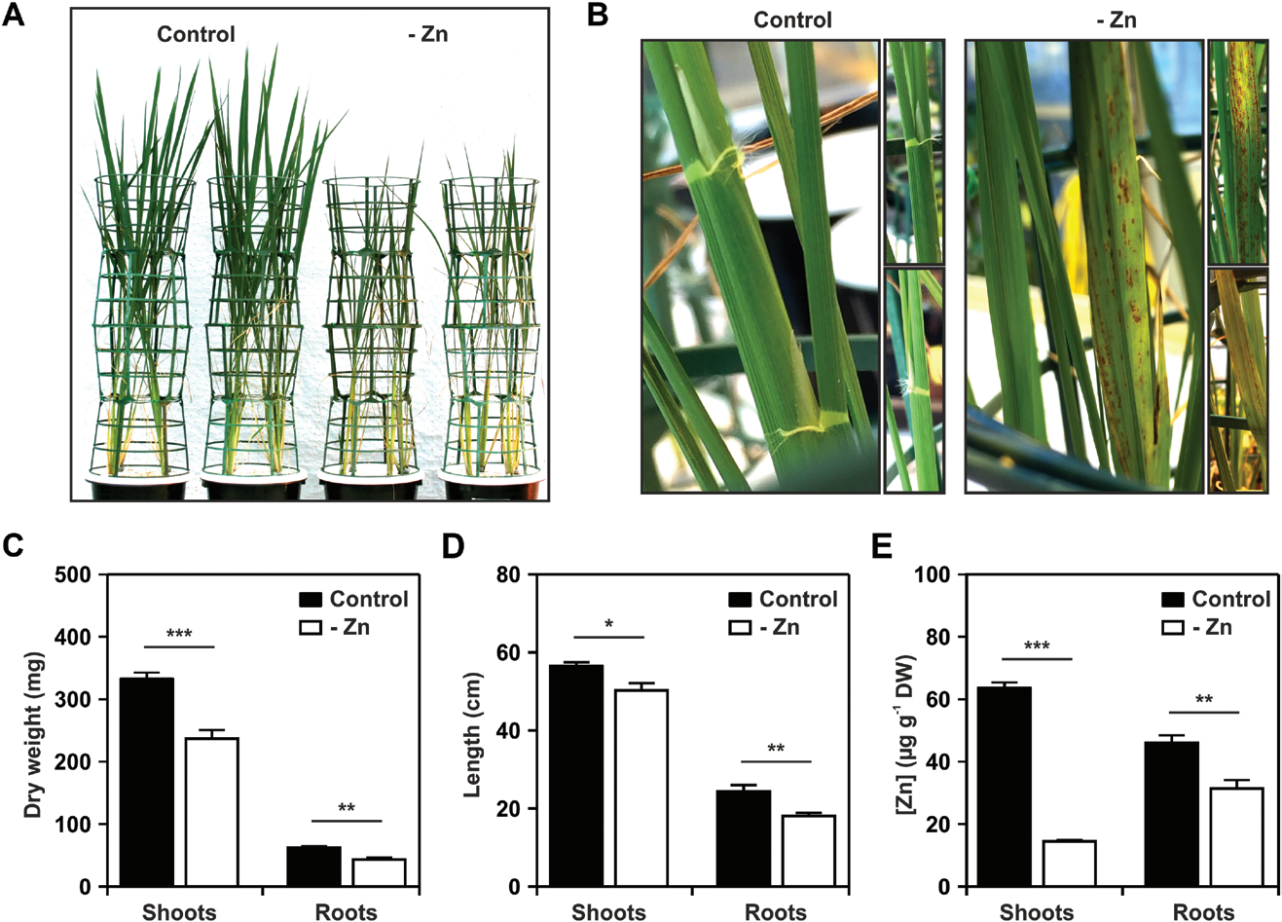
Phenotypic analysis of hydroponically grown rice plants in response to Zn supply. (A) Twelve-week-old rice plants grown with control and Zn-deficient (–Zn) nutrient solution. (B) Detail showing chlorosis and necrosis development with –Zn. Shoot and root dry weight (C), shoot and root length (D), and shoot and root Zn concentration (E), in 4-week-old rice plants grown in control and –Zn nutrient solution. Data are presented as means ±SE (*n*≥5 plants). Statistically significant differences between control and –Zn were determined by Student *t*-test (*P<0.05, **P<0.01, ***P<0.001).

To further our understanding of F-bZIP function in monocot Zn homeostasis, we analyzed rice plants grown hydroponically with control or Zn-deficient conditions. In 12-week-old plants, there were visible phenotypic differences between control and Zn deficiency treatments, with the latter displaying reduced growth and development of leaf chlorosis and necrosis (Fig. 3A, B). Four-week-old plants already displayed a reduction in both shoot and root dry weight, and shoot and root length, under Zn deficiency (Fig. 3C, D). As expected, element analysis revealed a significant reduction in the concentration of Zn in shoots and roots of Zn deficiency-grown plants. This reduction was more pronounced in shoots than in roots (Fig. 3E).

**Fig. 4.**
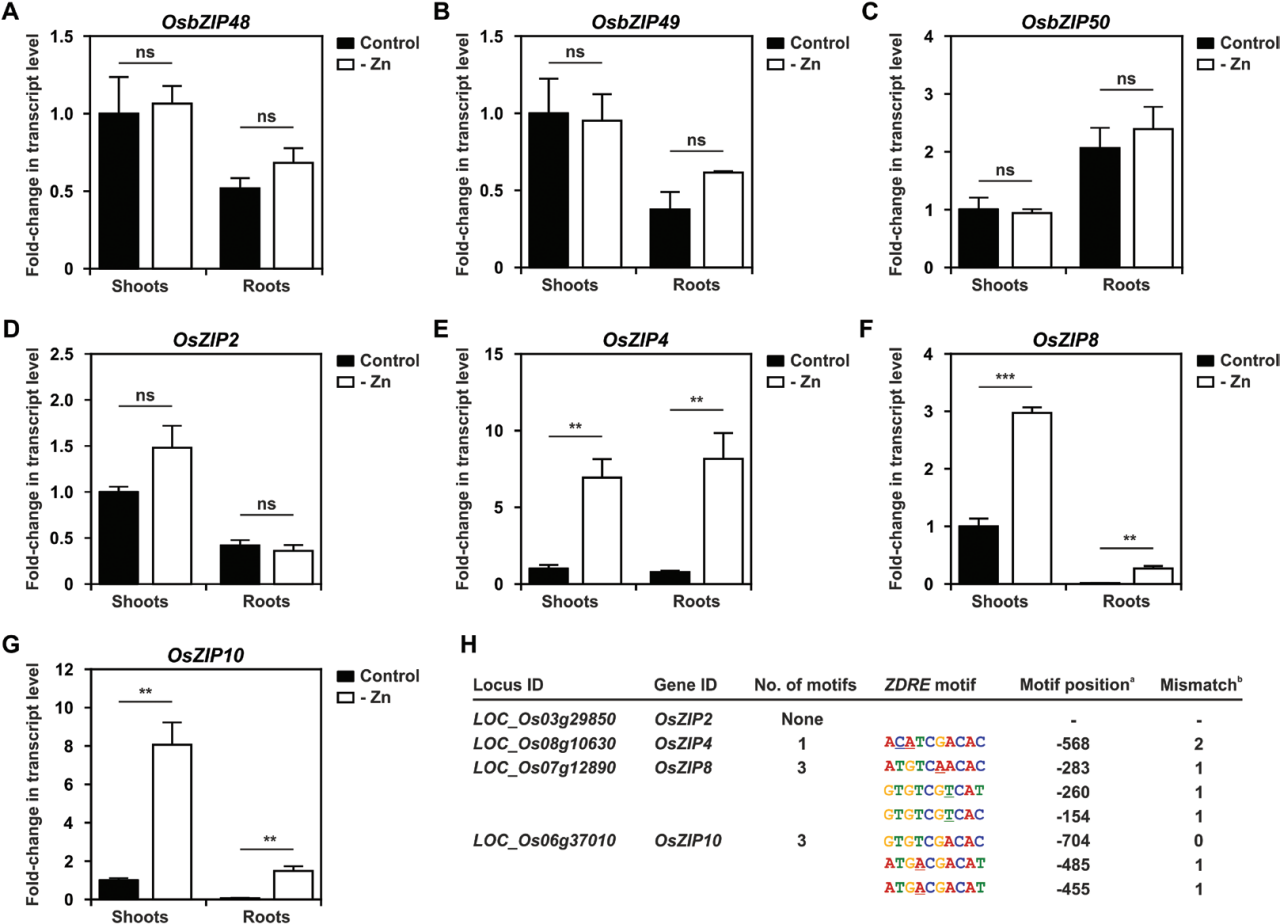
Gene expression analysis of 4-week-old hydroponically grown rice plants in response to Zn supply. Shoot and root transcript levels of (A) *OsbZIP48*, (B) *OsbZIP49*, (C) *OsbZIP50*, (D) *OsZIP2*, (E) *OsZIP4*, (F) *OsZIP8*, and (G) *OsZIP10*, grown with control or –Zn nutrient solution. Bars represent mean fold change in the transcript level of three biological replicates ±SE. Statistically significant differences between control and –Zn conditions were determined by Student *t*-test (**P<0.01, ***P<0.001; ns indicates not significant). (H) Analysis of the number and position of detected *ZDRE* motifs in the promoter of each gene. ^a^Position of the first nucleotide of the *ZDRE* motif in relation to the start codon. ^b^Mismatches from the *ZDRE* consensus sequence (RTGTCGACAY).

Next, we analyzed the transcript levels of the rice *F-bZIP* genes, *OsbZIP48*, *OsbZIP49*, and *OsbZIP50*. Our previous *in silico* analysis indicated that *OsbZIP49* has a lower expression level than *OsbZIP48* and *OsbZIP50* (Castro *et al.*, 2017). Here we show that *OsbZIP48* and *OsbZIP49* are expressed more in shoots than in roots, whereas *OsbZIP50* has the opposite expression pattern (Fig. 4A–C). None of the *F-bZIP* genes showed variation in transcript levels between control and Zn deficiency in shoots or roots (Fig. 4A–C). We also determined the expression of four rice *ZIP* family member genes, *OsZIP2*, *OsZIP4*, *OsZIP8*, and *OsZIP10*. *OsZIP2* expression was not significantly Zn deficiency responsive (Fig. 4D), whereas *OsZIP4*, *OsZIP8*, and *OsZIP10* expression was significantly induced under Zn deficiency in both shoots and roots (Fig. 4E–G). We analyzed the promoter region of these rice *ZIP* genes to identify the presence of the *ZDRE* motif. In the *OsZIP2* promoter, no *ZDRE* was found, whereas in *OsZIP8* and *OsZIP10*, three *ZDRE* copies were identified in each promoter region, with one mismatch allowed. In the *OsZIP4* promoter, a *ZDRE*-like motif, with two mismatches (A**CA**TCGACAC), was detected (Fig. 4H).

### Complementation analysis in the Arabidopsis *bzip19/23* background

The Arabidopsis *bzip19/23* double mutant is characterized by a Zn deficiency-hypersensitive phenotype (Assunção *et al.*, 2010). Here, the complementation lines of each rice F-bZIP member (*OsbZIP48*, *OsbZIP49*, and *OsbZIP50*) in the *bzip19/23* background were analyzed. To generate these heterologous complementation lines, we stably transformed the *bzip19/23* double mutant with each of the rice *F-bZIP* gene cDNAs under control of the constitutive CaMV *35S* promoter and containing a C-terminal CFP-HA fusion (i.e. *bzip19/23-*OE*Os*48, *bzip19/23-*OE*Os*49, and *bzip19/23-*OE*Os*50). The transcript level of the expressed rice gene in each line was verified, and a western blot with anti-HA confirmed the expected protein molecular weight for each rice bZIP-CFP-HA expressed protein in the heterologous complementation lines (Supplementary Fig. S3).

**Fig. 5.**
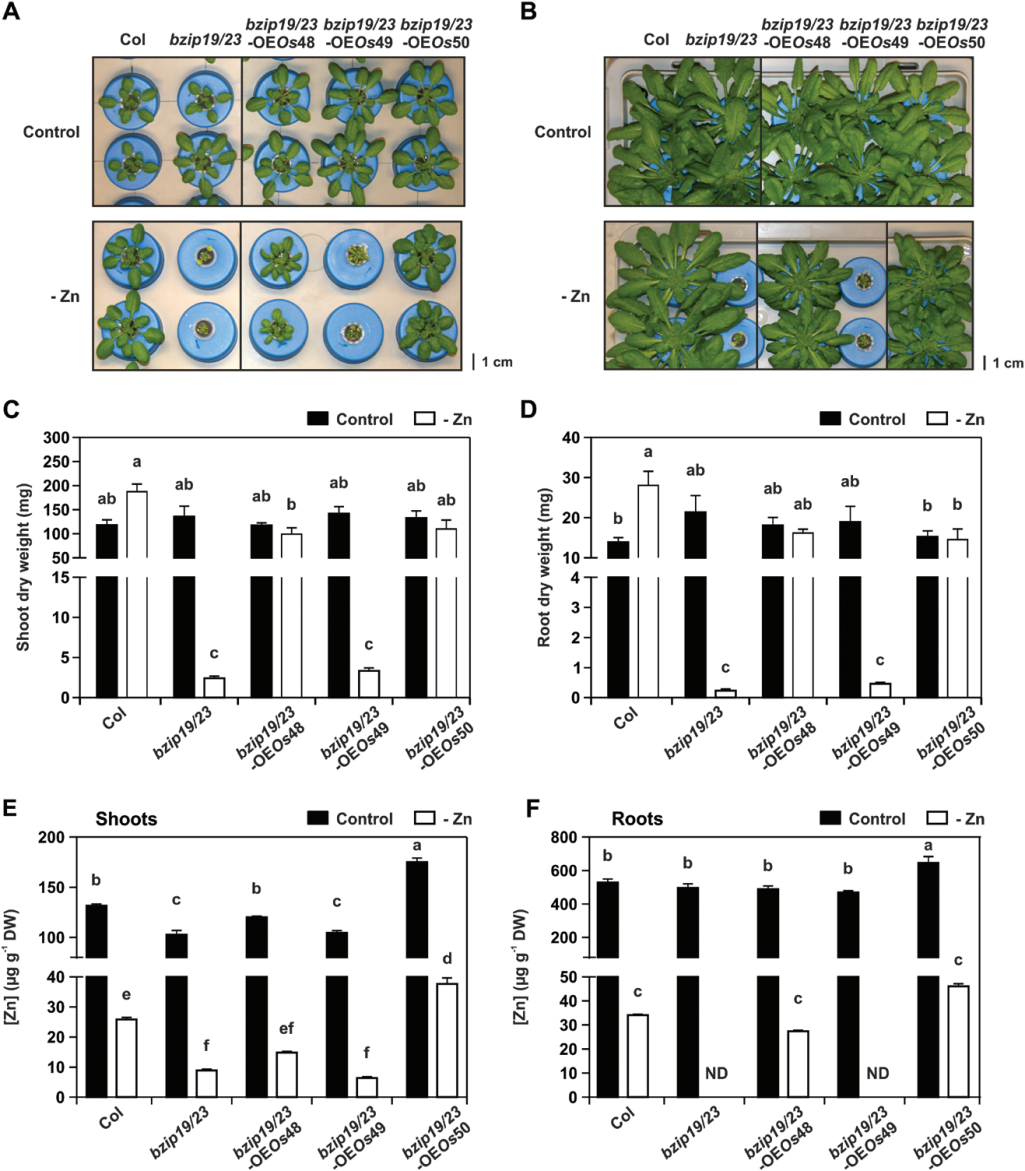
Complementation study with Arabidopsis *bzip19/23*-OE*Os*48, *bzip19/23*-OE*Os*49, and *bzip19/23*-OE*Os*50 lines, the wild type (Col), and the *bzip19/23* double mutant, grown in hydroponics with control or Zn-deficient (–Zn) nutrient solution. (A) Phenotype of 4-week-old plants and (B) 8-week-old plants. (C) Shoot and (D) root dry weight, and (E) shoot and (F) root Zn concentration, of 8-week-old plants grown under control (filled bar) or –Zn (open bar) conditions. Data are represented as means ±SE (*n*=4 plants for all treatments, except *bzip19/23* and *bzip19/23*-OE*Os*49 at –Zn with *n*=6). Different letters indicate significant differences (*P*<0.05) after one-way ANOVA followed by Tukey’s post-hoc test.

To test the complementation of the *bzip19/23* mutant, the *bzip19/23-*OE*Os*48, *bzip19/23-*OE*Os*49, and *bzip19/23-*OE*Os*50 lines were grown in hydroponics with control or Zn-deficient conditions. The analysis showed that *bzip19/23*-OE*Os*48 and *bzip19/23*-OE*Os*50 lines complemented the *bzip19/23* mutant phenotype under Zn deficiency, conferring phenotypes comparable with that of the wild-type. The *bzip19/23*-OE*Os*49 line, on the other hand, showed severe growth impairment under Zn deficiency, comparable with the *bzip19/23* mutant (Fig. 5A). The plants were allowed to grow for 8 weeks, during which the Zn deficiency-hypersensitive phenotype of the *bzip19/23* mutant and *bzip19/23*-OE*Os*49 lines became more evident (Fig. 5B). This was in agreement with the shoot and root tissue dry weight data (Fig. 5C,D). The lines were also grown with control or Zn-deficient MS medium, with *bzip19/23*-OE*Os*48 and *bzip19/23*-OE*Os*50 seedlings complementing the *bzip19/23* mutant, whereas the performance of *bzip19/23*-OE*Os*49 seedlings was slightly better than that of the *bzip19/23* mutant line (Supplementary Fig. S4).

The analysis of Zn concentration in the 8-week-old hydroponically grown plants showed that under control conditions, the shoot and root Zn concentration of the *bzip19/23*-OE*Os*48 line was not significantly different from that of the wild type, whereas the shoot concentration of the *bzip19/23* mutant and *bzip19/23*-OE*Os*49 lines was significantly lower. Interestingly, the shoot Zn concentration of the *bzip19/23*-OE*Os*50 line was significantly higher, with an increase to ~1.3 that of the wild type (Fig. 5E, F). Under Zn deficiency, the roots harvested from the *bzip19/23* mutant and *bzip19/23*-OE*Os*49 plants were extremely small (<0.5 mg DW), in line with the severity of the Zn deficiency phenotype (Fig. 5B), and were not included in the analysis. The Zn concentration in shoots of the *bzip19/23* mutant and *bzip19/23*-OE*Os*49 lines was significantly lower than in the wild type, and the *bzip19/23*-OE*Os*48 and *bzip19/23*-OE*Os*50 lines, with the latter having a significantly higher Zn concentration than the wild type (Fig. 5E, F; Supplementary Fig. S7, S8).

### Subcellular localization in response to Zn supply

**Fig. 6.**
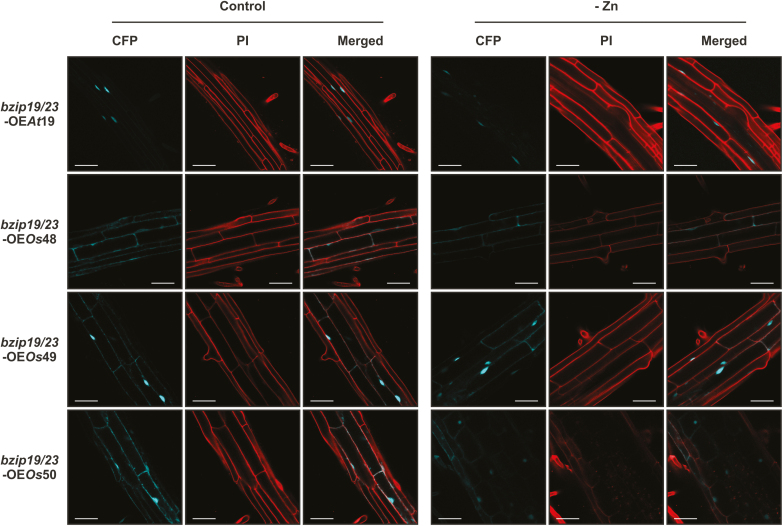
Subcellular localization analysis of OsbZIP48–CFP, OsbZIP49–CFP, and OsbZIP50–CFP fusion proteins in roots of 10-day-old seedlings of Arabidopsis *bzip19/23*-OE*Os*48, *bzip19/23*-OE*Os*49, and *bzip19/23*-OE*Os*50 lines, respectively, grown with control or Zn-deficient (–Zn) MS medium. The nuclear-localized AtbZIP19–CFP is used for comparison. Emissions of CFP and PI were visualized with CLSM. Two to three seedlings of three independently transformed T_3_ homozygous lines of *bzip19/23*-OE*Os*48, *bzip19/23*-OE*Os*49, and *bzip19/23*-OE*Os*50 lines were analyzed. Scale bars=50 μm.

To investigate the subcellular localization of the rice F-bZIP proteins and address whether it is affected by cellular Zn status, we analyzed seedlings of the *bzip19/23*-OE*Os*48, *bzip19/23*-OE*Os*49, and *bzip19/23*-OE*Os*50 lines, grown with control or Zn-deficient medium. The fluorescence of the C-terminal CFP fluorophore was visualized with CLSM. The fluorescent signal for the three rice F-bZIP protein fusions (OsbZIP48–CFP, OsbZIP49–CFP, and OsbZIP50–CFP) localized in the cell nucleus and also in the cytosol (Fig. 6). The analysis also revealed that the subcellular localization of the three rice F-bZIPs was identical between seedlings grown under control conditions and Zn deficiency (Fig. 6). These results were consistent between the three independently transformed lines analyzed for each complementation line. In addition, we performed a localization analysis using transient expression in *Nicotiana benthamiana* leaves with each rice *F-bZIP* cDNA under control of the constitutive CaMV *35S* promoter and containing an N-terminal green fluorescent protein (GFP) fluorophore. We found similar results for the three F-bZIP proteins (Supplementary Fig. S5).

### Expression analysis of bZIP19/23 target genes in the heterologous complementation lines

**Fig. 7.**
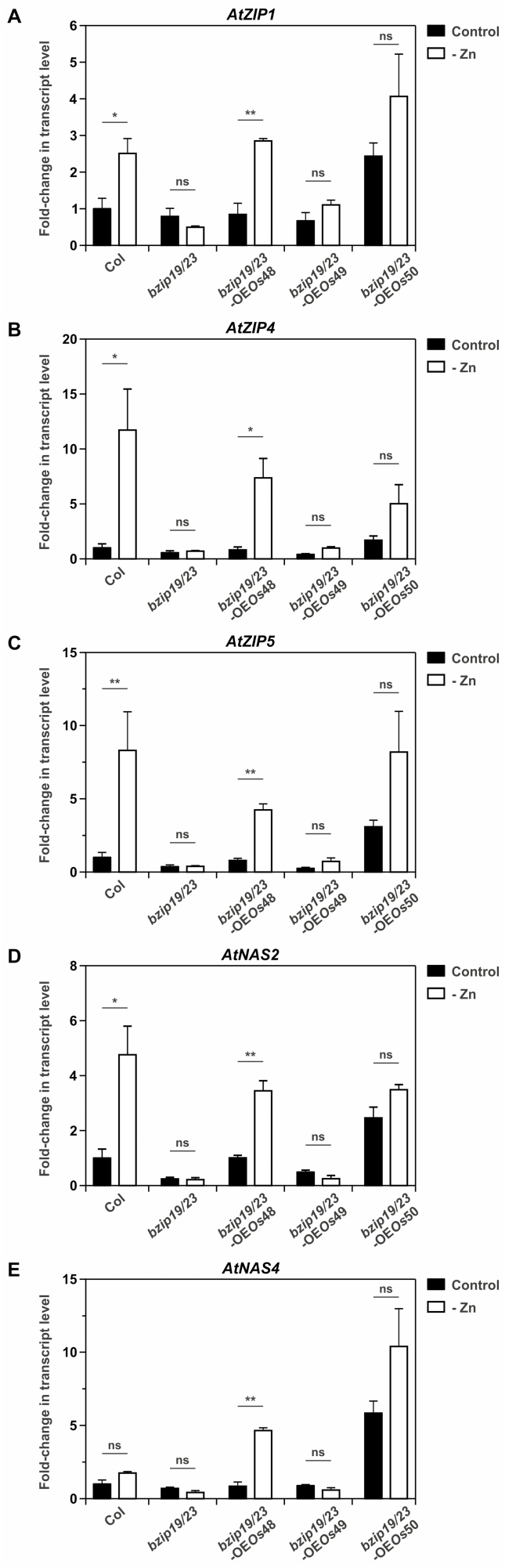
Transcript level profiles of bZIP19 and bZIP23 target genes in 14-day-old seedlings of wild-type Arabidopsis (Col), the *bzip19/23* double mutant, and *bzip19/23*-OE*Os*48, *bzip19/23*-OE*Os*49, and *bzip19/23*-OE*Os*50 lines, grown with control (filled bars) or Zn-deficient (–Zn, open bars) MS medium. Real-time quantitative RT–PCR was used to determine the transcript levels of (A) *ZIP1*, (B) *ZIP4*, (C) *ZIP5*, (D) *NAS2*, and (E) *NAS4*. Bars represent mean fold change in transcript level of three biological replicates ±SE. Data correspond to one independently transformed T_3_ homozygous line of *bzip19/23*-OE*Os*48, *bzip19/23*-OE*Os*49, and *bzip19/23*-OE*Os*50 lines. Data on the second independently transformed lines are shown in Supplementary Fig S6. Statistically significant differences between control and –Zn were determined by Student *t*-test (*P<0.05, **P<0.01; ns, indicates not significant).

To further analyze the rice F-bZIP complementation lines, we investigated the expression profiles of a subset of the Arabidopsis bZIP19 and bZIP23 target genes, namely *ZIP1*, *ZIP4*, *ZIP5*, *NAS2*, and *NAS4* (Assunção *et al.*, 2010) in seedlings of *bzip19/23*-OE*Os*48, *bzip19/23*-OE*Os*49, and *bzip19/23*-OE*Os*50 lines grown under control conditions or Zn deficiency (Fig. 7; Supplementary Fig. S6). The transcript level profiles were overall comparable between the *bzip19/23*-OE*Os*48 line and wild type, showing a significant induction of gene expression under Zn deficiency, indicating that the Group 1 OsbZIP48 protein was capable of complementing the Arabidopsis *bzip19/23* mutant (Fig. 7A–E). With regard to Group 2 rice F-bZIPs, the *bzip19/23*-OE*Os*49 line showed a transcript level profile that was overall comparable with that of the *bzip19/23* mutant—displaying gene expression levels under both Zn conditions that collectively support the absence of functional complementation (Fig. 7A–E). Conversely, under Zn deficiency, the expression pattern in the *bzip19/23*-OE*Os*50 line was overall comparable with that of the wild type and *bzip19/23*-OE*Os*48, in agreement with the complementation analysis. Most significantly, under control conditions, *bzip19/23*-OE*Os*50 displayed a high transcript level for all genes analyzed, surpassing not only the null mutant levels but also those of the wild type (Fig. 7A–E).

### Element analysis in soil-grown plants

**Fig. 8.**
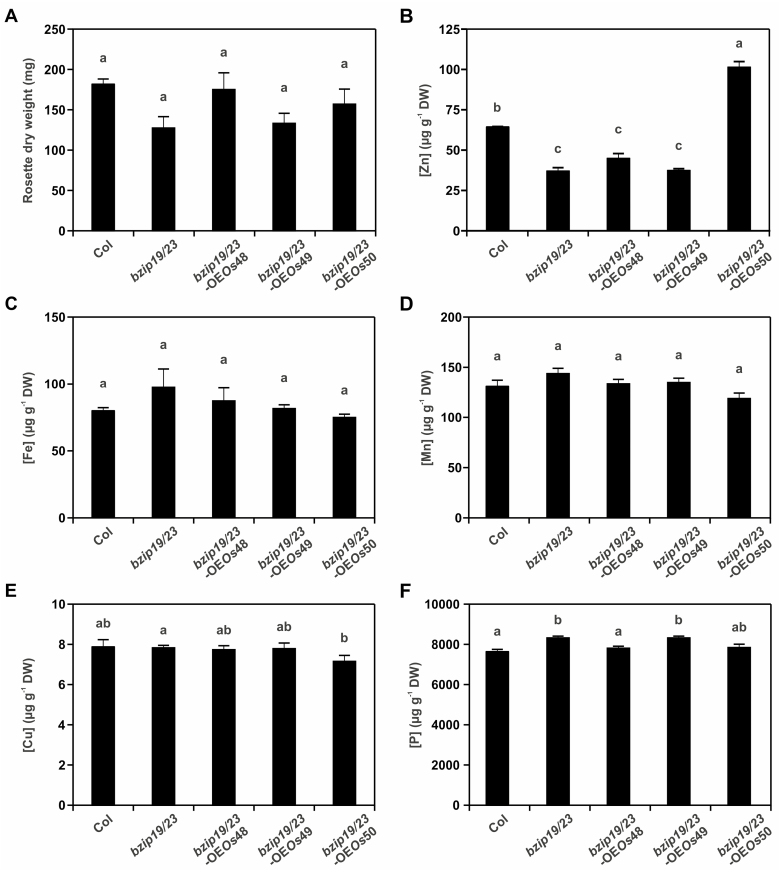
Element analysis of shoots of 6-week-old plants of the Arabidopsis wild type (Col), *bzip19/23* double mutant, and *bzip19/23*-OE*Os*48, *bzip19/23*-OE*Os*49, and *bzip19/23*-OE*Os*50 lines, grown on soil. Data correspond to one independently transformed T_3_ homozygous lines. Data on second and third independently transformed lines are shown in Supplementary Figs S7 and S8. Bars represent (A) shoot dry weight, (B) Zn concentration, (C) Fe concentration, (D) Mn concentration, (E) Cu concentration, and (F) P concentration. Data are represented as means ±SE (*n*=4 plants). Different letters indicate significant differences (*P*<0.05) after one-way ANOVA followed by Tukey’s post-hoc test.

Finally, we also analyzed the shoot Zn concentration of soil-grown plants, which displayed a similar pattern when compared with the hydroponically grown plants under control conditions (Fig. 8A). Here, the *bzip19/23*-OE*Os*50 line showed a significantly higher Zn concentration, with an increase to ~1.5 that of the wild type (Fig. 8A). In addition, the analysis of other elements, namely Fe, Mn, Cu, and P, showed that their concentrations in shoots did not differ strongly between the wild type, the mutant, and the complementation lines (Fig. 8B–F; Supplementary Fig. S8).

## Discussion

### Phylogenetic analysis of monocot F-bZIPs

In this study, we identified and functionally characterized F-bZIP members of rice ssp. *japonica*, namely LOC_Os06g50310, LOC_Os01g58760, and LOC_Os05g41540, addressing their role in the Zn deficiency response. The classification of the bZIP family in Arabidopsis comprises 13 groups (designated A–M) (Jakoby *et al.*, 2002; Dröge-Laser *et al.*, 2018) and this classification has been extended to all major lineages of green plants in an evolutionary analysis (Corrêa *et al.*, 2008). In this classification, the above-mentioned rice F-bZIPs correspond to OsbZIP48, OsbZIP49, and OsbZIP50, respectively (Corrêa *et al.*, 2008). A different classification for rice bZIP family members comprises 11 groups (designated I–XI), in which the same rice F-bZIPs correspond to OsbZIP53, OsbZIP44, and OsbZIP7, respectively (Nijhawan *et al.*, 2008). We adopted the former classification system.

Our previous phylogenetic analysis of F-bZIP homologs across land plants suggested a monophyletic origin prior to seed plant evolution, where branching out of two clades (Group 1 and Group 2) was observed (Castro *et al.*, 2017). Here, our phylogenetic and synteny analysis of monocot F-bZIP homologs supports the previously described emergence of F-bZIP Groups 1 and 2 associated with seed plant differentiation (Fig. 2; Supplementary Fig. S2). Rice bZIP48 belongs to Group 1, together with Arabidopsis bZIP19 and bZIP23 proteins, the central regulators of the Zn deficiency response (Assunção *et al.*, 2010). The wheat TraesCS7D02G518100/TabZIPF1-7DL that complements the Arabidopsis *bzip19/23* mutant (Evens *et al.*, 2017) is also in Group 1. Rice bZIP49 and bZIP50 belong to Group 2, together with Arabidopsis bZIP24, involved in salt stress regulation, and with no significant role in the Zn deficiency response (Yang *et al.*, 2009; Lilay *et al*., 2018). The analysis also indicates an enrichment of Group 2 F-bZIP genes in monocots, in relation to Group 1, that contrasts with the observed pattern in eudicots, where several species lost Group 2 F-bZIP homologs (Castro *et al.*, 2017). Previously, we suggested that Group 1 F-bZIPs conserve a function in the Zn deficiency response regulation, whereas Group 2 F-bZIPs appear more prone to gene loss or expansion events that might lead to neo- or subfunctionalization (Castro *et al.*, 2017).

### Role of rice F-bZIPs in the zinc deficiency response

The role of the three rice F-bZIPs in the Zn deficiency response was investigated. The expression profiles of *OsbZIP48*, *OsbZIP49*, and *OsbZIP50* in rice roots and shoots indicate that these genes are not responsive to Zn deficiency (Fig. 4), suggesting that a Zn-dependent activity of the transcription factors is probably not regulated at the transcriptional level. This is in line with results obtained for the Arabidopsis F-bZIP transcription factors (Lilay *et al*., 2018). In wheat and barley, some of the identified *F-bZIP* homologs showed gene expression induction under Zn deficiency (Evens *et al.*, 2017; Nazri *et al.*, 2017) and, for wheat, it was suggested that Zn deficiency induction of some *F-bZIP* genes would allow a sustained adaptive response under prolonged deficiency (Evens *et al.*, 2017). Interestingly, among the wheat and barley F-bZIP homologs, those reported to complement the Arabidopsis *bzip19/23* mutant, or to show *in vitro* binding to the *ZDRE* motif (i.e. TraesCS7D02G518100/TabZIPF1-7DL, HvbZIP56, and HvbZIP62), displayed only a small transcript induction, if any, upon Zn deficiency (Evens *et al.*, 2017; Nazri *et al.*, 2017).

The heterologous expression of the three rice F-bZIPs in Arabidopsis and *N. benthamiana* indicated a nuclear and also cytosolic localization (Fig. 6; Supplementary Fig. S5), in line with the results obtained for the barley HvbZIP56 expressed in Arabidopsis (Nazri *et al.*, 2017), whereas AtbZIP19 and AtbZIP23, also with a 35S promoter, were shown to be nuclear localized (Inaba *et al.*, 2015; Lilay *et al*., 2018). Our results additionally indicate that the cellular Zn status is not involved in the subcellular targeting of rice F-bZIP proteins, comparable with what was reported for Arabidopsis F-bZIPs (Lilay *et al*., 2018), suggesting that a Zn-dependent modulation of the activity of these transcription factors involves other mechanisms.

Several members of the rice ZIP family were characterized and shown to be involved in Zn uptake and transport (Bashir *et al.*, 2012). We analyzed the expression of four rice *OsZIP* genes as Zn deficiency markers for our hydroponically grown rice plants, and verified the presence of *ZDRE* motifs in their promoter region (Fig. 4). Previously, the analysis of predicted orthologs of the Arabidopsis bZIP19/23 target *ZIP* genes in land plant species found *ZDRE* motifs in the promoters and identified a conserved substitution in the motif (i.e. RTG**W**CGACAY) (Castro *et al.*, 2017). Here, we identified three promoter *ZDRE* motifs (RTGWCGACAY) in the Zn deficiency-induced *OsZIP10*, and three *ZDRE* variants, with one mismatch, in the Zn deficiency-induced *OsZIP8* (Lee *et al.*, 2010). For the strongly Zn deficiency-induced *OsZIP4*, functionally characterized as a Zn transporter (Ishimaru *et al.*, 2005), no *ZDRE* motif was found but a *ZDRE* variant, with two mismatches, was detected, which deserves further analysis. Conversely, in the non-Zn deficiency-induced *OsZIP2*, similarly to its close homolog *AtZIP2* (Assunção *et al.*, 2010; Ishimaru *et al.*, 2011), no motif was found in the promoter region. Overall, our data indicate an association between Zn deficiency-induced rice *ZIP* genes and the presence of promoter *ZDRE* motifs, as previously reported for *ZIP* genes from wheat, barley, and other land plant species (Castro *et al.*, 2017; Evens *et al.*, 2017; Nazri *et al.*, 2017), supporting the conservation of the Zn deficiency response regulatory mechanism.

Our findings additionally show that OsbZIP48 and OsbZIP49 bind not only to the *ZDRE* motif *in vitro* but also to a *ZDRE*-containing *AtZIP4* promoter fragment in a Y1H assay. The yeast screening did not identify *OsbZIP50* possibly because, as we show, *OsbZIP50* is expressed more in roots than in shoots, and the rice cDNA library was enriched for shoot transcripts. *OsbZIP48* and *OsbZIP49* showed the opposite expression pattern—more expressed in shoots than in roots. This was also observed between Arabidopsis *bZIP19* and *bZIP23* expression patterns and might be relevant for the individual role of each transcription factor in the Zn deficiency response (Lilay *et al*., 2018).

### OsbZIP48 is a functional homolog of AtbZIP19 and AtbZIP23

The role of the three rice F-bZIPs in the Zn deficiency response was further investigated by complementation analysis in the Arabidopsis *bzip19/23* double mutant background, namely *bzip19/23*-OE*Os*48, *bzip19/23*-OE*Os*49, and *bzip19/23*-OE*Os*50 lines (Figs 5, 7, 8). Our observations indicate that Group 1 OsbZIP48 is a functional homolog of AtbZIP19 and AtbZIP23. The *bzip19/23*-OE*Os*48 line complemented the *bzip19/23* mutant, restoring Zn concentration levels in the plant similar to the wild type. In addition, the analysis of AtbZIP19/23 target gene expression showed Zn deficiency induction in the *bzip19/23*-OE*Os*48 line, similarly to the wild type, and to the Arabidopsis bZIP19 and bZIP23 complementation lines (*bzip19/23*-OE19 and *bzip19/23*-OE23) (Lilay *et al*., 2018). A wild-type expression pattern of Zn deficiency-responsive *AtZIP* target genes was also reported in the *bzip19/23* complementation lines overexpressing barley *HvbZIP56* and *HvbZIP62* (Nazri *et al.*, 2017). According to our results, a Zn-dependent regulation of rice F-bZIPs is unlikely to occur at the transcriptional level, and cellular Zn status does not seem to interfere with their subcellular targeting. Furthermore, the expression profile of the target genes in the *bzip19/23*-OE*Os*48 line suggests that cellular Zn status can repress or limit the activity of OsbZIP48. This is consistent with previous findings from Arabidopsis bZIP19 and bZIP23, supporting that Zn deficiency sensing is required for the activity of these transcription factors (Lilay *et al*., 2018).

The *bzip19/23*-OE*Os*49 line did not complement the *bzip19/23* mutant and displayed its severe Zn deficiency phenotype, with the Zn shoot concentration in line with the reported critical deficiency level below 15–20 µg g^−1^ DW (Marschner, 1995). This was consistent with a similar pattern of target gene expression between *bzip19/23*-OE*Os*49 and the *bzip19/23* mutant. Group 2 OsbZIP49 is a protein lacking much of the N-terminal region typically found in F-bZIPs which very probably disturbs its function, explaining the lack of complementation. The conservation of the bZIP domain, with the basic region and leucine-zipper domain, involved in DNA binding and directing dimerization, respectively (Vinson *et al.*, 2006), is consistent with our EMSA and Y1H results indicating binding of OsbZIP49 to the *ZDRE* motif.

The Group 2 OsbZIP50 complemented the *bzip19/23* mutant in the *bzip19/23*-OE*Os*50 line, which is interesting considering that the Group 2 Arabidopsis bZIP24 has not been associated with the regulation of Zn deficiency response. The most remarkable difference between the *bzip19/23*-OE*Os*50 line and the wild-type and *bzip19/23*-OE*Os*48 lines is the increased shoot Zn concentration found in *bzip19/23*-OE*Os*50 plants grown under Zn-sufficient conditions while the concentration of other elements was not significantly affected. Moreover, the expression of the target genes was consistently higher in the *bzip19/23*-OE*Os*50 line, under control conditions, than in the wild type or *bzip19/23*-OE*Os*48 lines. This suggests that the enhanced shoot Zn concentration in *bzip19/23*-OE*Os*50 is caused by higher expression of, at least, the analyzed genes. The Arabidopsis bZIP19 and bZIP23 target genes are transcriptionally activated upon Zn deficiency, with Zn sufficiency limiting or repressing their activation (Lilay *et al*., 2018). Our results suggest that the effect of Zn sufficiency in repressing target gene activation by OsbZIP50 is less efficient than in AtbZIP19, AtbZIP23, and OsbZIP48. The increased expression of AtbZIP19/23 target genes and the enhanced shoot Zn concentration in the *bzip19/23-*OE*Os*50 line under Zn sufficiency provide a novel perspective suggesting that the Zn-dependent activity of the F-bZIP transcription factors could be modulated to improve plant Zn acquisition.

We provide a detailed monocot F-bZIPs phylogenetic analysis that supports the branching of plant F-bZIPs into Group 1 and Group 2. Our results show that the rice F-bZIP transcription factors play a role in the Zn deficiency response, with Group 1 OsbZIP48 being a functional homolog of Arabidopsis bZIP19 and bZIP23, supporting a conservation of this regulatory mechanism in land plants. More knowledge on the regulators and mechanisms of the Zn deficiency response in crops will contribute to effectively address Zn use efficiency and Zn biofortification, and develop plant-based strategies to address the problems of Zn deficiency in soils and in human diets.

## Supplementary data

Supplementary data are available at *JXB* online.

Fig. S1. Multiple sequence alignment of F-bZIP members.

Fig. S2. Pairwise collinearity analysis between monocots F-bZIP genes.

Fig. S3. Transcript level profiles and western blot analysis.

Fig. S4. Complementation analysis.

Fig. S5. Subcellular localization analysis.

Fig. S6. Transcript level profiles of the *bzip19/23-*OE*Os*48, *-*OE*Os*49, and *-*OE*Os*50 second lines.

Fig. S7. Element analysis of the *bzip19/23-*OE*Os*48 and *-*OE*Os*49 second lines.

Fig. S8. Element analysis of the *bzip19/23-*OE*Os*50 second and third lines.

Table S1. Primers used for cloning.

Table S2. Primers used in real-time quantitative RT–PCR analysis.

Table S3. Clones sequenced in the Y1H screening.

Table S4. Monocot F-bZIPs sequences and IDs.

eraa115_suppl_supplementary_figures_S1-S8_tables_S1-S2

eraa115_suppl_supplementary_table_S3

eraa115_suppl_supplementary_table_S4
