# Supplementary material for: Rice F-bZIP transcription factors regulate the zinc deficiency response
Source: J Exp Bot. 2020 Mar 5;71(12):3664–77. doi: 10.1093/jxb/eraa115 (PMC7307843; doi:10.1093/jxb/eraa115)
Supplement: eraa115_suppl_supplementary_figures_S1-S8_tables_S1-S2 [file eraa115_suppl_supplementary_figures_s1-s8_tables_s1-s2.pdf]

Supplementary data

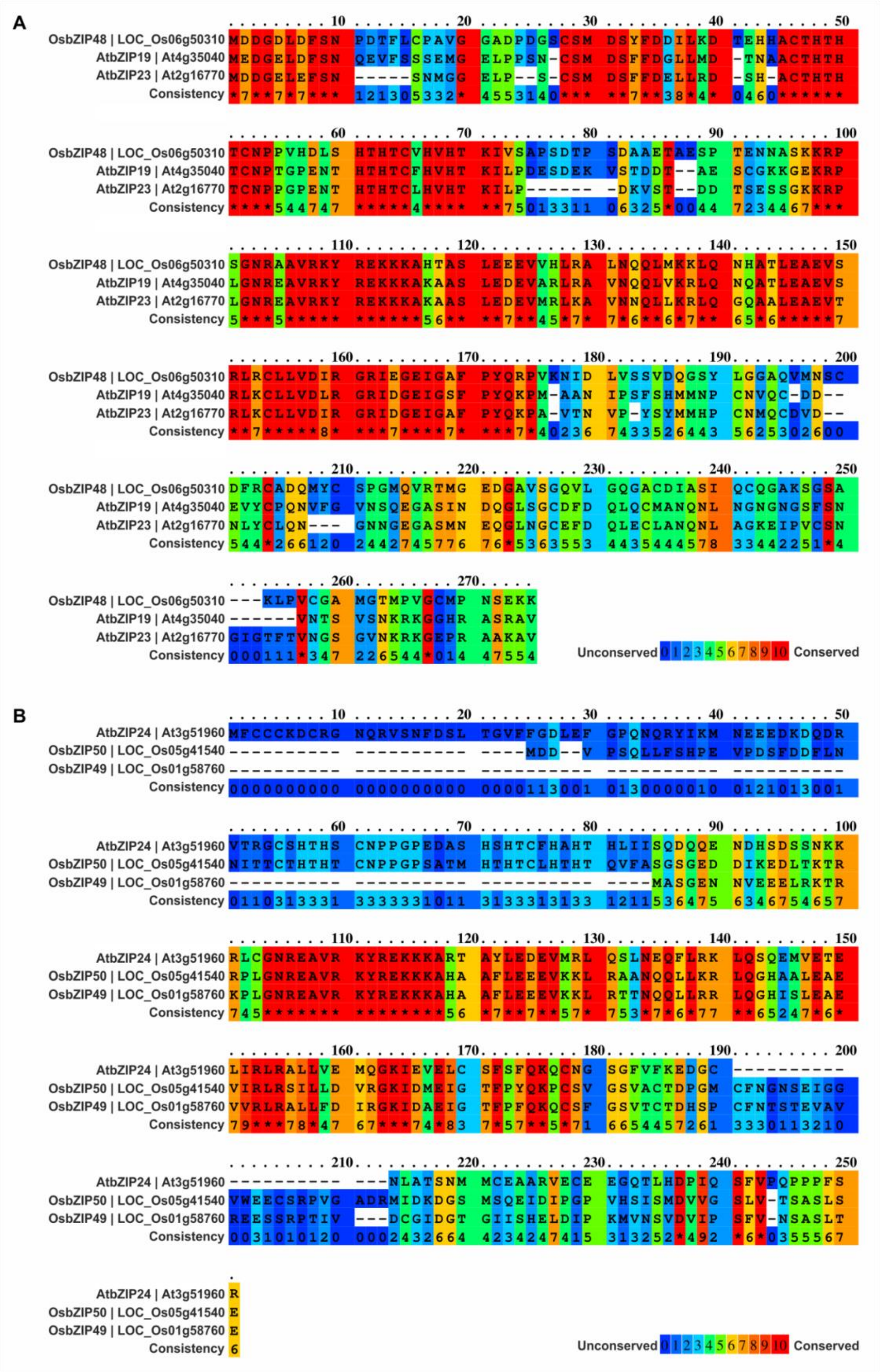

Figure S1. Multiple sequence alignment of Group 1 (**A**), and Group 2 (**B**) F-bZIP members from Arabidopsis and rice (*Oryza sativa spp. japonica*). Amino acid consistency is classified from 0 (unconserved) to 10 (conserved). The characteristic Cys/His-rich motif and bZIP domain are highly conserved.

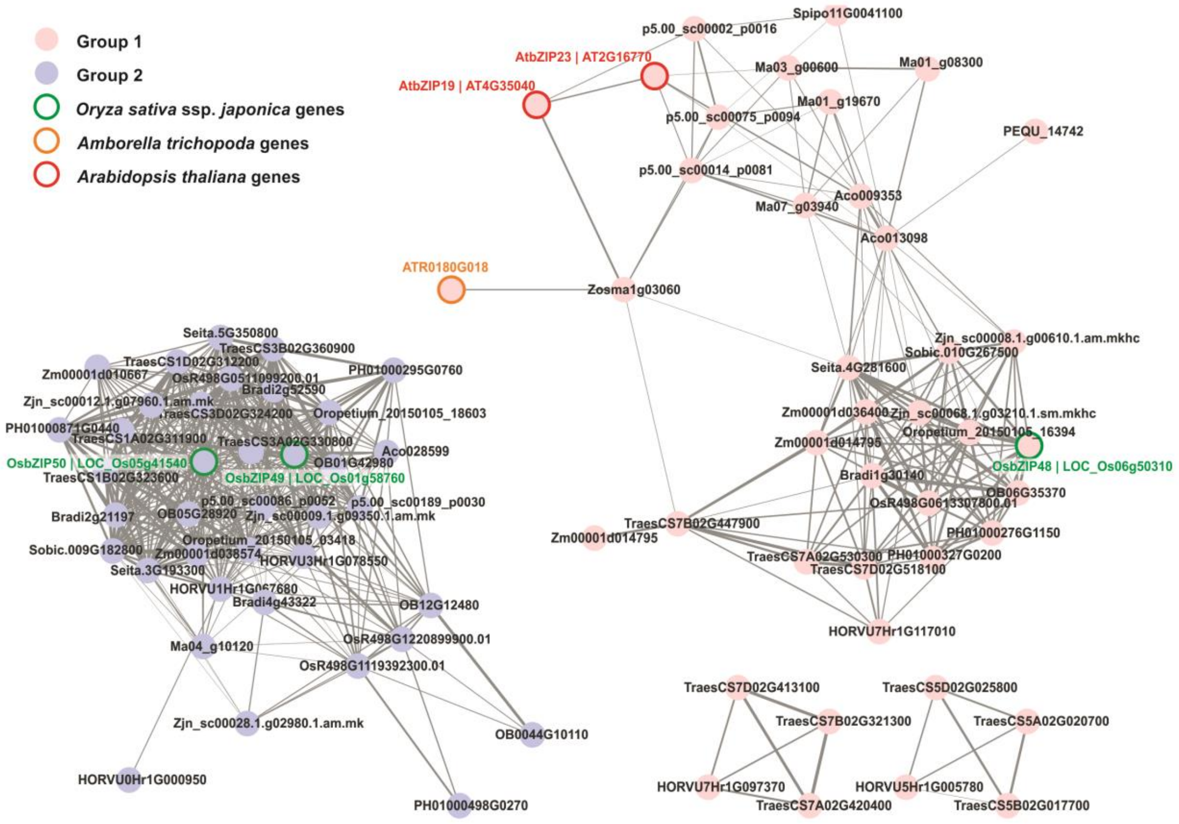

Figure S2. Pairwise collinearity analysis between all F-bZIP genes in a set of selected species within Monocots (*Ananas comosus*, *Brachypodium distachyon*, *Elaeis guineensis*, *Hordeum vulgare*, *Musa acuminata*, *Oropetium thomaeum*, *Oryza brachyantha*, *Oryza sativa* ssp. *indica*, *Oryza sativa* ssp. *japonica*, *Phalaenopsis equestris*, *Phyllostachys edulis*, *Setaria italica*, *Sorghum bicolor*, *Spirodela polyrhiza*, *Triticum aestivum*, *Zea mays*, *Zostera marina*, and *Zoysia japonica* ssp. *Nagirizaki*), and Dicots (*Arabidopsis thaliana* and *Amborella trichopoda*). Plaza Monocots 4.5 was used to interrogate each gene for the presence of a paralog anchored in a syntenic block in any of the other species (regardless of whether it was a Group 1 or -2 F-bZIP). Strength of collinearity is given by the number of anchorpoints present within two syntenic blocks. A network was constructed to spatially summarize the presence and strength of the collinearity between all F-bZIPs. The network represents genes (nodes) identified as belonging to syntenic blocks. Edges represent the estimated collinearity strength between gene pairs. Genes where no collinearity was observed were removed. Paralogs that diverged more recently will tend to conserve collinearity between the genomic regions they are anchored on. This serves as a survey to infer of the evolution of gene family members that is complementary to phylogenetic analysis, by looking at paralogs within their genomic context. This analysis showed no collinearity between Group 1 and 2 members, but strong collinearity within each Group.

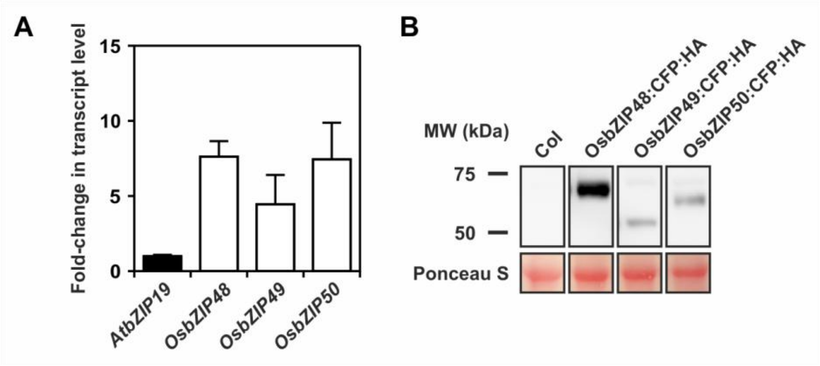

Figure S3. (**A**) Transcript level profiles of *OsbZIP48*, *OsbZIP49* and *OsbZIP50* using real-time quantitative RT-PCR, in 14-day-old seedlings of *bzip19/23*-OE*Os*48, *bzip19/23*-OE*Os*49 or *bzip19/23*-OE*Os*50 lines, respectively, grown in MS medium. The relative transcript levels were expressed against the *AtbZIP19* in Arabidopsis wild-type seedlings. Bars represent mean fold-change in transcript level of three biological replica ±SE (**B**) Western blot analysis of bZIP48-CFP-HA, bZIP49-CFP-HA and bZIP50-CFP-HA fusion proteins expressed in 10-day-old seedlings of wild-type (Col), *bzip19/23*-OE*Os*48, *bzip19/23*-OE*Os*49 and *bzip19/23*-OE*Os*50 lines grown in MS medium. Total protein per sample (20 µg) was verified with Ponceau S staining, and anti-HA primary antibody was used. Expected protein molecular weight for bZIP48-CFP-HA, bZIP49-CFP-HA and bZIP50-CFP-HA are 59.72, 48.75 and 55.08 KDa, respectively.

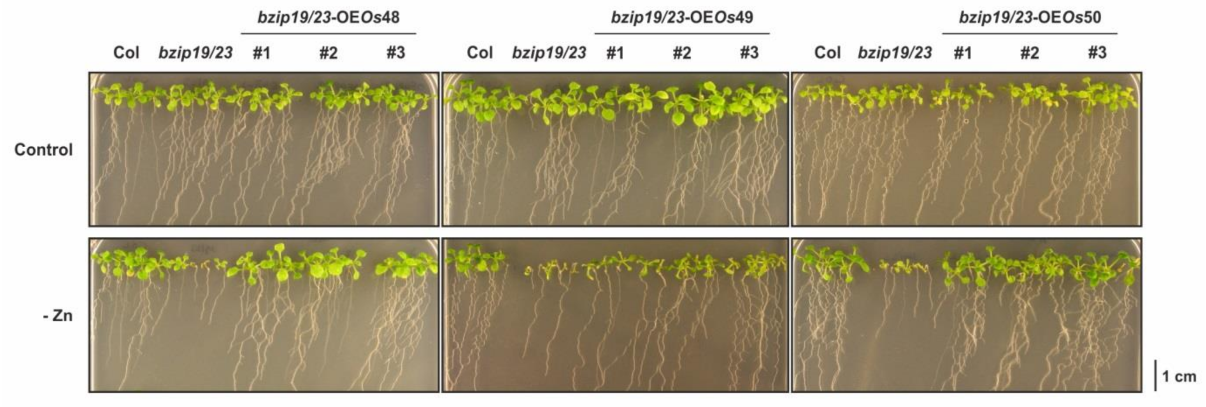

Figure S4. Seedlings of 14-day-old Arabidopsis wild-type (Col), *bzip19/23* double mutant, and three independently transformed T3 homozygous lines of *bzip19/23*-OE*Os*48, *bzip19/23*-OE*Os*49 and *bzip19/23*-OE*Os*50, grown with control or Zn-deficient (-Zn) MS medium.

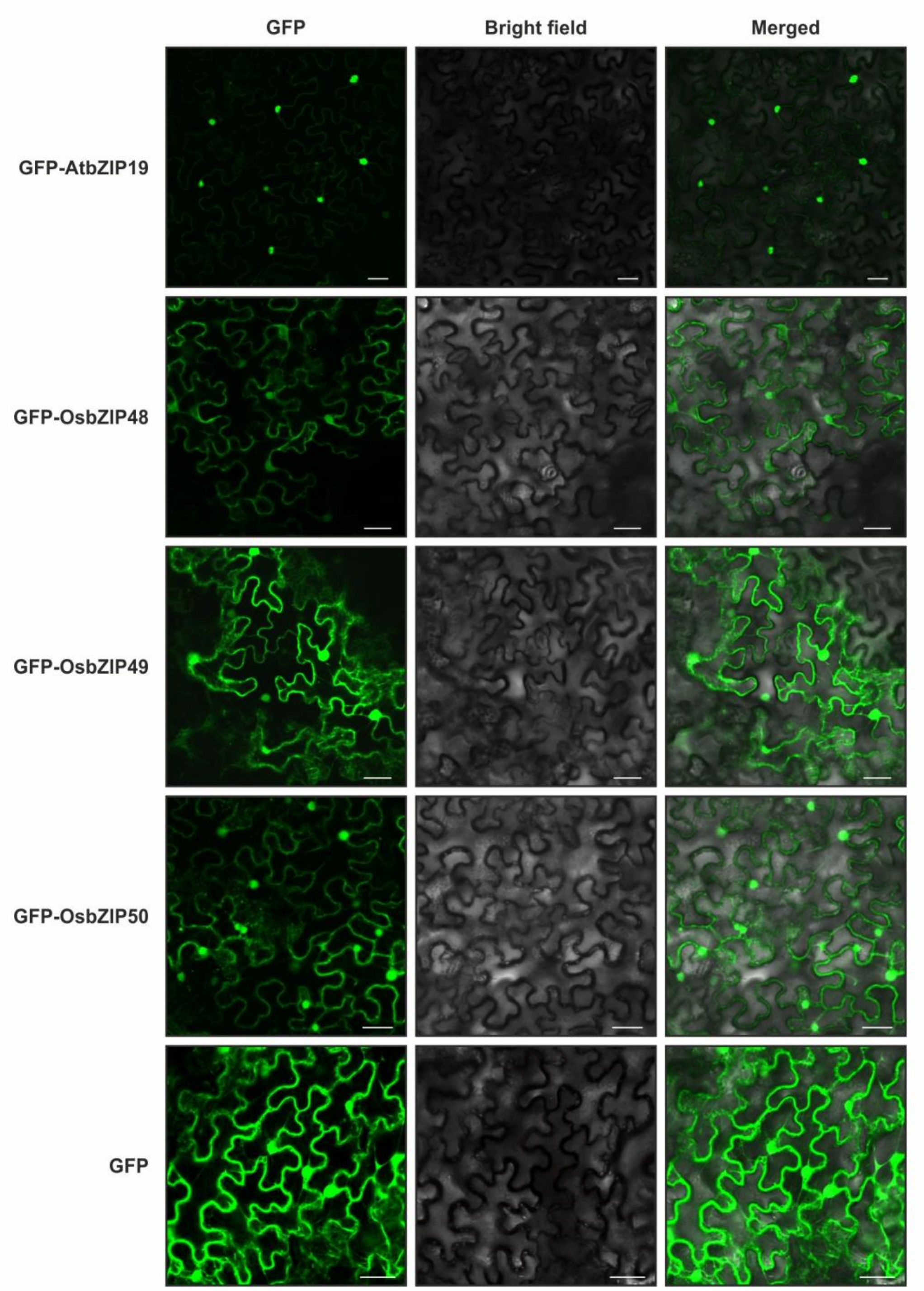

Figure S5. Subcellular localization analysis of GFP-OsbZIP48, GFP-OsbZIP49 and GFP-OsbZIP50 fusion proteins transiently expressed in *Nicotiana benthamiana* leaves. The nuclear localized GFP-AtbZIP19 is used for comparison. Emissions of GFP and bright-field (transmission) were visualized with confocal laser scanning microscopy (CLSM) for GFP-AtbZIP19, GFP-OsbZIP48, GFP-OsbZIP49, GFP-OsbZIP50 and GFP. Scale bars = 40 μm.

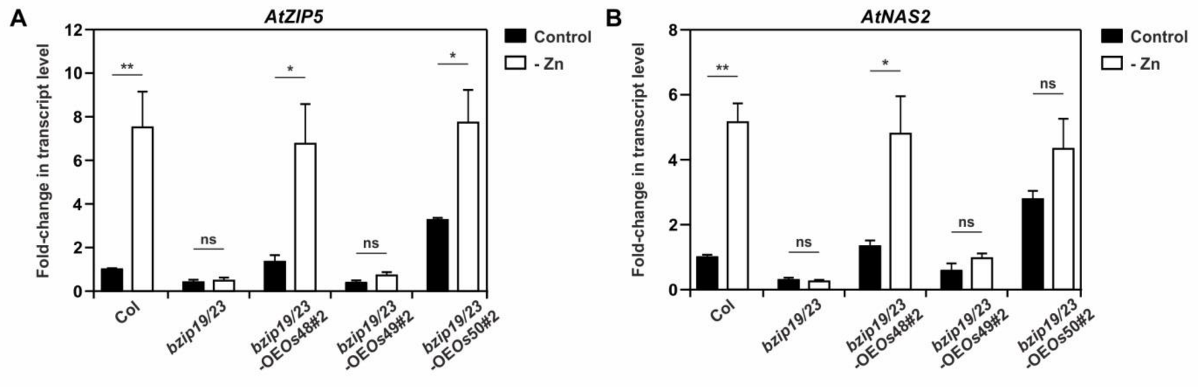

Figure S6. Transcript level profiles of bZIP19 and bZIP23 target genes in 14-day-old seedlings of wild-type Arabidopsis (Col), *bzip19/23* double mutant, and second independently transformed T3 homozygous lines of *bzip19/23*-OE*Os*48, *bzip19/23*-OE*Os*49 and *bzip19/23*-OE*Os*50, grown on control (dark bars) or Zn-deficient (-Zn, open bars) MS medium. Real-time quantitative RT-PCR was used to determine the transcript levels of (**A**) *ZIP5* and (**B**) *NAS2*. Bars represent mean fold-change in transcript level of three biological replica ±SE. Statistically significant differences between control and –Zn were determined by Student t-test (* $p < 0.05$, ** $p < 0.01$, ns means not significant).

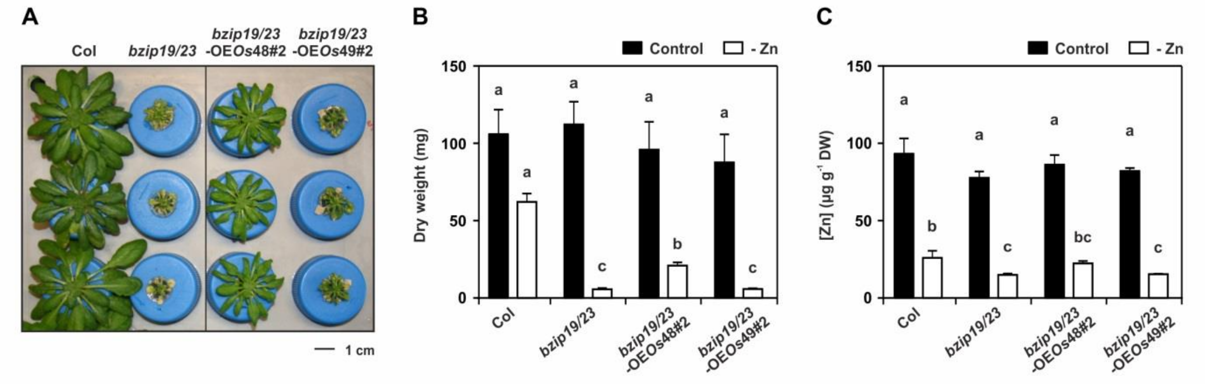

Figure S7. Complementation study with Arabidopsis second independently transformed T3 homozygous lines of *bzip19/23*-OE*Os*48 and *bzip19/23*-OE*Os*49, and wild-type (Col) and *bzip19/23* double mutant, grown in hydroponics. (**A**) Phenotype of the different lines (**B**) shoot dry weight, and (**C**) shoot Zn concentration, of 6-week-old plants grown at control (black bar) or –Zn (open bar). Data are represented as means ±SE (n=4 plants for all treatments, except *bzip19/23* and *bzip19/23*-OE*Os*49 at –Zn with n=6). Different letters indicate significant differences (p < 0.05) after one-way ANOVA followed by Tukey's post-hoc test.

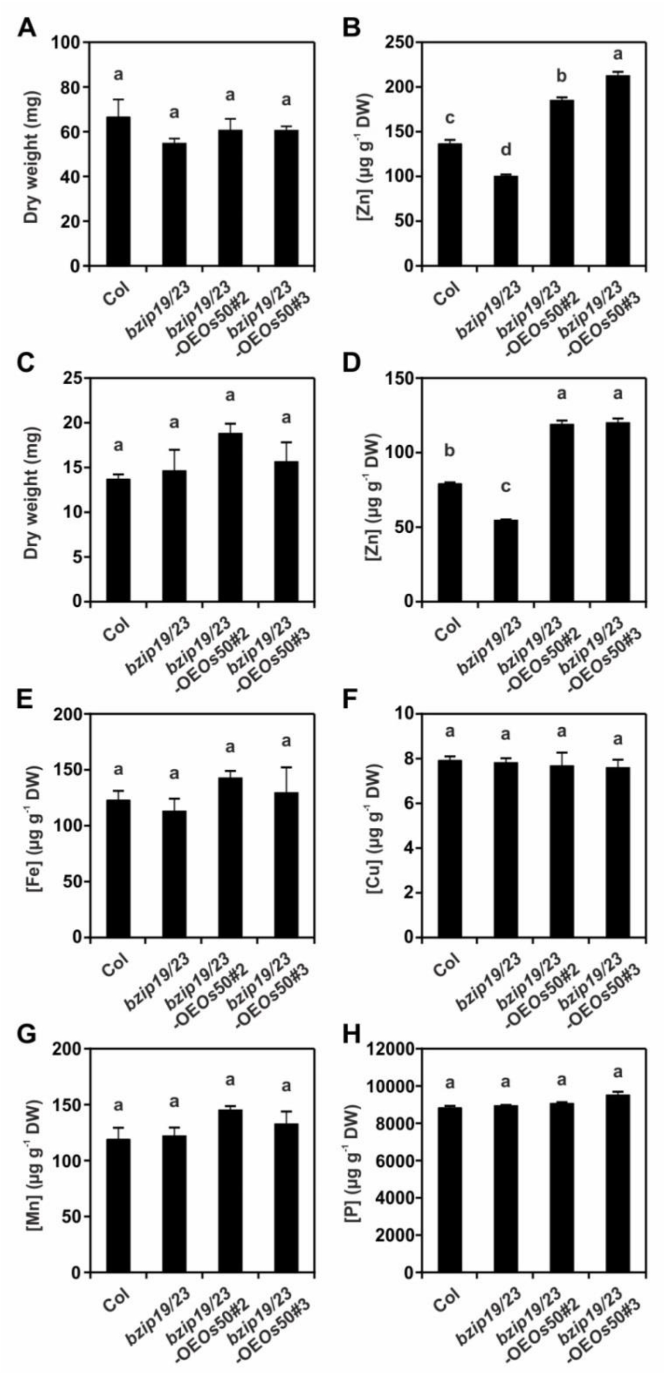

Figure S8. Element analysis of shoots of Arabidopsis wild-type (Col), *bzip19/23* double mutant, and second and third independently transformed T3 homozygous lines of *bzip19/23*-OE*Os*50. (**A**) Shoot dry weight and (**B**) Zn concentration in 6-week-old plants grown in hydroponics with control nutrient solution. (**C**) Shoot dry weight, (**D**) Zn concentration, (**E**) Fe concentration, (**F**) Cu concentration, (**G**) Mn concentration, and (**H**) P concentration of 4-week-old plants grown on soil. Data are represented as means ±SE (n=4 plants). Different letters indicate significant differences ($p < 0.05$) after one-way ANOVA followed by Tukey's post-hoc test.

Table S1. Forward (F) and reverse (R) primers, containing *Not*I (F) and *Asc*I (R) restriction enzyme sites (bold), used for cloning the CDS of rice *OsbZIP48, OsbZIP49 and OsbZIP50.*

**Primer name**	**Sequence 5'-3'**
OsbZIP48-F	GG**GCGGCCGC**CATGGATGACGGGGACCTCGATTTC
OsbZIP48-R	AT**GGCGCGCC**CTTTCTTTTCAGAATTTGGCATAC
OsbZIP49-F	AT**GCGGCCGC**CATGGCTTCTGGTGAAAACAATGTTG
OsbZIP49-R	AA**GGCGCGCC**CTTCAGTCAATGAGGCAGAATTC
OsbZIP50-F	AT**GCGGCCGC**CATGGACGATGTCCCAAGTCAATTG
OsbZIP50-R	AA**GGCGCGCC**CCTCAGATAGAGAAGCAGAAGTC

Table S2. Forward (F) and reverse (R) primers used in real-time quantitative RT-PCR analysis to determine transcript levels of rice *OsbZIP48*, *OsbZIP49* and *OsbZIP50* and reference gene *OseEF-1α*, and Arabidopsis *AtZIP1*, *AtZIP4*, *AtZIP5*, *AtNAS2*, *AtNAS4* and reference gene *AtACT2*.

**Primer name**	**Sequence 5'-3'**
OsbZIP48 F	TGTGACTTTCGATGTGCCGA
OsbZIP48 R	TGGGAGCTTTGCAGATCCAG
OsbZIP49 F	GGTGGTGAGGTTAAGAGCCC
OsbZIP49 R	GCTCGTGCGAAATAATGCCA
OsbZIP50 F	GCGCAAGTATCGGGAGAAGA
OsbZIP50 R	CCGGACATCGAGTAGGATGC
OseEF-1α F	TGGTGACCAAGATCGACAGA
OseEF-1α R	GCATCACCGTTCTTGAGGA
OsZIP2 F	AAATGGCAACGCCTCTGACC
OsZIP2 R	CTCGAAGACGGAGTGGAAGC
OsZIP4 F	GGTTGTCTCCCAGGTTCTG
OsZIP4 R	TGATCACTGTTGCTTTCGCC
OsZIP8 F	TGATCATTGGCATCTCCCTCG
OsZIP8 R	TTGAACTTCGCCTGCACGATG
OsZIP10 F	CATGTGGTTGTCTCGCAGATTC
OsZIP10 R	GTGGAATGAGAGGGCAGCAA
AtACT2 F	CTAAGCTCTCAAGATCAAAGGCTTA
AtACT2 R	ACTAAAACGCAAAACGAAAGCGGTT
AtZIP1 F	GGACACACACATGGTTCGAC
AtZIP1 R	GATAGTGCAGCCATGAGTGG
AtZIP4 F	GATCTTCGTCGATGTTCTTTGG
AtZIP4 R	TGAGAGGTATGGCTACACCAGCAGC
AtZIP5 F	CGGGATTGTTGGCGTGGAAT
AtZIP5 R	CCAAGACCCTCGAAGCATTG
AtNAS2 F	CGACGTGGTTAATTCGGTGG
AtNAS2 R	CGCGTGGACCTTAGAGCAAT
AtNAS4 F	GTCGTTCTTGCCTCTTCCCA
AtNAS4 R	GAGCAGCCAAGAACACAACG

Supplementary protocols

Protein extraction and immunoblotting

Ten-day-old seedlings of wild-type, *bzip19/23*-OE*Os*48, *bzip19/23*-OE*Os*49 and *bzip19/23*-OE*Os*50 lines grown in control or -Zn MS medium were grinded in a microtube in liquid nitrogen, with the help of polypropylene pestles. Protein extracts were obtained by adding extraction buffer (50 mM Tris; 150 mM NaCl; 0.2 % (v/v) Triton X-100) supplemented with Complete Protease Inhibitor Cocktail (Roche), as manufacturer instructions. Following incubation for 1 h at 4 °C with agitation, microtubes were centrifuged for 2 × 30 min at 16,000 *g*. The supernatant was recovered and stored at −80 °C. Protein was spectrophotometrically quantified using Bradford Reagent (Sigma-Aldrich) (Bradford 1976). Total protein (20 µg per sample) was separated electrophoretically in a Mini-PROTEAN TGX Precast gel using Mini-PORTEAN Cell (Bio-Rad) apparatus. Proteins were transferred to Trans-Blot Turbo Midi PVDF Transfer Packs membranes using Trans-Blot Turbo Transfer System (Bio-Rad) following standard protocol. PVDF membranes were blocked overnight at 4 ºC in blocking solution (5% dry milk powder in PBST). The primary antibody Anti-HA (ab9110, Abcam) was added in a 1:4000 dilution and incubated for 2 h. Membranes were washed three times, at constant rotation for 10 min, with approximately 10 mL of PBST. The membranes were incubated for 1 h with the secondary antibody Anti-Rabbit (IgG-peroxidase, A6154, Sigma) in a 1:10,000 dilution in blocking solution. The membrane was washed as previously described and developed using the Immun-Star WesternC Kit (Bio-Rad) and a Chemidoc Touch Imaging System (Bio-Rad) for image acquisition. As protein equal loading reference, membranes were incubated for 15 min with Ponceau S solution to stain total protein.

Subcellular localization analysis in *Nicotiana benthamiana* leaves

The *pCaMV35S::GFP-bZIP48, pCaMV35S::GFP-bZIP49* and *pCaMV35S::GFP-bZIP50* constructs for transient transformation of *Nicotiana benthamiana* leaves were generated by *in vitro* site-directed recombination from the pENTR™/D-TOPO constructs into pMCD43 Gateway vector (Curtis, 2003), carrying a *CaMV35S* promoter and a N-terminal GFP fluorophore, using LR Clonase™ II Enzyme Mix (Invitrogen). Transformation into *A. tumefaciens* and *Nicotiana benthamiana* leaves were performed as described by Lilay *et al.* (2019). For the subcellular localization analysis, three plants were infiltrated per construct and were visualized 4-5 days after infiltration using a laser scanning confocal microscope Leica TCS SP5-X (Leica Microsystems) with a HCX PL APO 20x /0.70 water objective. White light laser at 480 nm was used for GFP excitation. The emission settings were between 500-580 nm for GFP, 631-721 nm for far-red with transmission (bright field) also recorded.
